# C781, a *β*-Arrestin Biased Antagonist at Protease-Activated Receptor-2 (PAR2), Displays in vivo Efficacy Against Protease-Induced Pain in Mice

**DOI:** 10.1016/j.jpain.2022.11.006

**Published:** 2022-11-20

**Authors:** Moeno Kume, Ayesha Ahmad, Stephanie Shiers, Michael D. Burton, Kathryn A. DeFea, Josef Vagner, Gregory Dussor, Scott Boitano, Theodore J. Price

**Affiliations:** * Department of Neuroscience and Center for Advanced Pain Studies, University of Texas at Dallas; † PARMedics Incorporated, Richardson, Texas; ‡ University of Arizona Bio5 Institute; § Asthma and Airway Disease Research Center, University of Arizona Heath Sciences; ¶ Department of Physiology, University of Arizona Heath Sciences

**Keywords:** C781, neutrophil elastase, nociception, PAR2, biased antagonism

## Abstract

Given the limited options and often harmful side effects of current analgesics and the suffering caused by the opioid crisis, new classes of pain therapeutics are needed. Protease-activated receptors (PARs), particularly PAR2, are implicated in a variety of pathologies, including pain. Since the discovery of the role of PAR2 in pain, development of potent and specific antagonists has been slow. In this study, we describe the in vivo characterization of a novel small molecule/peptidomimetic hybrid compound, C781, as a β-arrestin-biased PAR2 antagonist. In vivo behavioral studies were done in mice using von Frey filaments and the Mouse Grimace Scale. Pharmacokinetic studies were done to assess pharmacokinetic/pharmacodynamic relationship in vivo. We used both prevention and reversal paradigms with protease treatment to determine whether C781 could attenuate protease-evoked pain. C781 effectively prevented and reversed mechanical and spontaneous nociceptive behaviors in response to small molecule PAR2 agonists, mast cell activators, and neutrophil elastase. The ED_50_ of C781 (intraperitoneal dosing) for inhibition of PAR2 agonist (20.9 ng 2-AT)-evoked nociception was 6.3 mg/kg. C781 was not efficacious in the carrageenan inflammation model. Pharmacokinetic studies indicated limited long-term systemic bioavailability for C781 suggesting that optimizing pharmacokinetic properties could improve in vivo efficacy. Our work demonstrates in vivo efficacy of a biased PAR2 antagonist that selectively inhibits β-arrestin/MAPK signaling downstream of PAR2. Given the importance of this signaling pathway in PAR2-evoked nociception, C781 exemplifies a key pharmacophore for PAR2 that can be optimized for clinical development.

**Perspective:**

Our work provides evidence that PAR2 antagonists that only block certain aspects of signaling by the receptor can be effective for blocking protease-evoked pain in mice. This is important because it creates a rationale for developing safer PAR2-targeting approaches for pain treatment.

Chronic pain is a disease affecting about one-third of the population in both the United States and the United Kingdom.^43^ The cost of this public health problem in the United States is approximately 560 to 635 billion dollars per year in medical treatment and lost productivity.^29^ Current treatment options are limited and can have deleterious side effects, which have unfortunately contributed to a surge in opioid overdose deaths in the past 2 decades.^5,19,24^ In 2017, 1.7 million Americans suffered from prescription opioid-related substance abuse disorders and more than 47,000 people died from opioid overdose, resulting in the U.S. Department of Health and Human Services (HHS) declaring the opioid crisis a public health emergency.^10,11,60^ There is a clear need for new types of analgesics that are both safe and effective.

Nearly 20 years ago, protease-activated receptor-2 (PAR2) emerged as a potential pain target when it was observed that knockout mice have decreased pain sensitization in response to inflammation.^61^ Discovered in the 1990s, the PARs are a family of G-protein coupled receptors (GPCRs) that, unique among GPCRs, do not have a known endogenous ligand ^1^. Instead, endogenous and exogenous proteases cleave the extracellular portion of the receptor, exposing a tethered ligand that binds to the receptor and catalyzes downstream signaling cascades.^49^ PAR2 is implicated in a variety of pain types, including pain from inflammation, cancer and other injuries that cause immune cells to release proteases^35,36,41,51,58,63^ and also migraine headache.^25,33,63^ After irreversible proteolytic cleavage by proteases, such as those released by neutrophils and mast cells at sites of inflammation, the receptor can either signal on the cell membrane via the recruitment of G proteins or through *β*-arrestin signaling pathways either at the cell surface of endosomally after internalization.^30,34^ By acting on PAR2 and initiating these biochemical pathways, proteases are thought to sensitize sensory neurons and cause hyperalgesia.^4,15,41,44^ PAR2 activation in nociceptors can induce G_q_-coupled Ca^2+^ mobilization and *β*-arrestin/mitogen activated protein kinase (MAPK) activation. G_q_-coupled Ca^2+^ mobilization promotes sensitization of transient receptor potential (TRP) ion channels causing thermal hyperalgesia.^4^ We have shown, however, that mechanical hypersensitivity and hyperalgesic priming caused by PAR2 activation requires activation of MAPK signaling in nociceptors.^57^ Moreover, although PAR2 is expressed by many cell types, nociception induced by proteases is caused by activation of PAR2 on a very specific subset of nociceptors that also express receptors and neurotransmitters involved in itch.^26,30^

We have described a series of potent and specific PAR2 agonists and antagonists, including the PAR2 agonist 2-aminothiazo-4-yl-LIGRL-NH_2_ (2AT) and the PAR2 antagonist C391.^6–8,20^ We now introduce a novel, smaller, more drug-like PAR2 antagonist molecule: C781. Our previously published work describes the in vitro pharmacology of C781, identifying the compound as a biased antagonist of PAR2 that selectively blocks *β*-arrestin/MAPK signaling downstream of receptor activation.^54^ In this study, we evaluated the in vivo efficacy and PK of C781 in mouse pain models. We measured both mechanical and spontaneous nociceptive behaviors using von Frey filaments and the Mouse Grimace Scale (MGS), respectively, to determine the ability of C781 to block PAR2-mediated nociception.^27,30^ Our findings demonstrate that C781 is a potent PAR2 antagonist that effectively prevents nociceptive behaviors caused by 2AT, neutrophil elastase (a PAR2-cleaving enzyme acting through MAPK), and compound 48 of 80 (a mast cell degranulator), and reverses nociceptive behaviors caused by neutrophil elastase.

## Methods

### Animals

All experiments and procedures were approved by the Institutional Animal Care and Use Committee at University of Texas at Dallas and performed in accordance with the ARRIVE guidelines in accordance with *Journal of Pain* recommendations.^32^ Male and female ICR and C57BL6J bred in-house at the University of Texas at Dallas and weighing between 15 and 50 grams were used in this study. Animals were housed in a climate-controlled room set to 22°C with a 12-hour light/dark cycle and given food and water ad libitum. At the conclusion of behavioral experiments animals were euthanized by isoflurane overdose followed by cervical dislocation.

### Experimental Reagents

Compound 781 (C781) and 2-aminothiazo-4-yl-LIGRL-NH_2_ (2AT) were made using solid-phase synthesis, as previously described for 2AT and C781.^6,7,20,21,46,54^ For in vitro studies, neutrophil elastase (NE; cat# LS002274) and trypsin (cat# LS003703) were purchased from Worthington Biochemical Corporation, Lakewood New Jersey. NE was first diluted to 100 mM in Hanks Balanced Saline Solution (HBSS), pH 8.1. All subsequent dilutions of NE, trypsin, C781 or 2AT were in Minimal Essential Medium (MEM), pH 7.4. All experiments were conducted at 37°C and 5% CO_2_. For in vivo studies, NE was purchased from Elastin Products Company, Inc. (cat# SE563), compound 48/80 from Millipore Sigma (cat# C2313), *λ*-carrageenan from Sigma Aldrich (cat# 22049), and prostaglandin E2 (PGE2) from Cayman Chemical (cat# 14010). All compounds were dissolved in 0.9% saline. C781 was injected at doses indicated in the text into the intraperitoneal (i.p.) space using a 27G ½” needle. All other compounds were injected into the left hind paw using a glass Hamilton syringe (cat# 80901) and 30G ½” needle.

### Behavioral Methods

Experimenters were blinded to treatment groups in all experiments. Animals were habituated to suspended acrylic chambers with each individual chamber measuring 11.4 × 7.6 × 7.6 cm and a wire mesh bottom (1 cm^2^). Mechanical hypersensitivity was assessed using the manual von Frey hair test after spontaneous pain assessment using the Mouse Grimace Scale (MGS). This was done to assure that grimacing responses were not a result of mechanical stimulation.

MGS were determined using the MGS test as described by Langford and colleagues.^37^ After habituation, the experimenter observed the mice and assigned intensity ratings (0 = not present, 1 = moderately present, 2 = severe) to the facial action units relative to baseline. The 5 action units identified are 1) orbital tightening, 2) nose bulge, 3) cheek bulge, 4) ear position, and 5) whisker change. The scores for all 5 action units are averaged to obtain the MGS score for each time point. The MGS has been shown to be an accurate way of measuring spontaneous acute nociception in response to multiple types of stimuli, including PAR2 agonists.^2,38,40,59^

Hind paw withdrawal thresholds were calculated via the Dixon up-down method after applying calibrated von Frey filaments from Stoelting, Co. (cat# 58011) to the volar surface of the left hind paw.^12^ Increasing pressure was applied until the filament bent against the skin at a right angle and a positive response was recorded when the mouse showed nocifensive behaviors, such as rapid paw withdrawal, flicking, and/or licking of the stimulated paw. The maximum filament weight used was 2 g.

Hyperalgesic priming was determined after allowing animals to return to baseline levels and injecting the previously injected paw with 100 ng of PGE2 (Cayman Chemical, Ann Arbor, MI). The hyperalgesic priming model involves the use of a priming stimulus that will cause neuronal plasticity and trigger long-lasting sensitivity to subthreshold levels of inflammatory mediators, like PGE2, even after the initial injury has resolved.^31,53^

### Pharmacokinetics of C781

Pharmacokinetic studies were done at Sai Life Sciences Limited (Pune, India) under a contractual research agreement. Plasma concentration of C781 was measured following a single intraperitoneal injection of C781 in saline in male or female C57BL6 mice (8–12 weeks old, weighing between 20–35 g). Under light isoflurane anesthesia, blood samples (about 60 *μ*L each) were collected from the retro-orbital plexus at 5 minutes, 30 minutes, 1 hour, 2 hours, 4 hours, and 8 hours postinjection. Each time point consisted of 3 different blood samples, and plasma was immediately taken via centrifugation and stored at −70°C until analysis. Samples were processed for analysis by protein precipitation using acetonitrile and analyzed with fit-forpurpose LC/MS/MS method (LLOQ = 1.01 ng/mL). Calculations for pharmacokinetic parameters were done using the noncompartmental analysis tool of Phoenix WinNonlin software (Version 7.0).

### Data and Statistical Analysis

The data and statistical analysis comply with the recommendations on experimental design and analysis in pharmacology (Curtis et al, 2018). Sample sizes were estimated based on effect sizes observed in PAR2 sensory neuron-specific conditional knockout mice as reported previously.^27^ Animals were randomly assigned to groups and experiments were done blinded to treatment. All statistical tests were done using GraphPad Prism version 8.4.3 (GraphPad Software, Inc.). We assessed whether the data violated assumptions of parametric statistics via the Brown-Forsythe test and the Kolmogorov-Smirnov test when doing the analysis in GraphPad Prism. If the standard deviations were not significantly different and the data passed the normality test (alpha > .05), differences between groups were calculated using repeated measures 2-way ANOVAs with Dunnett’s multiple-comparison correction. If the data did not pass these tests, the Geisser-Greenhouse correction was used. All data are represented as mean ± SEM.

## Results

### Local Injection of C781 Attenuates Nociceptive Responses Induced by the PAR2 Agonist 2AT

**Figure 1.**
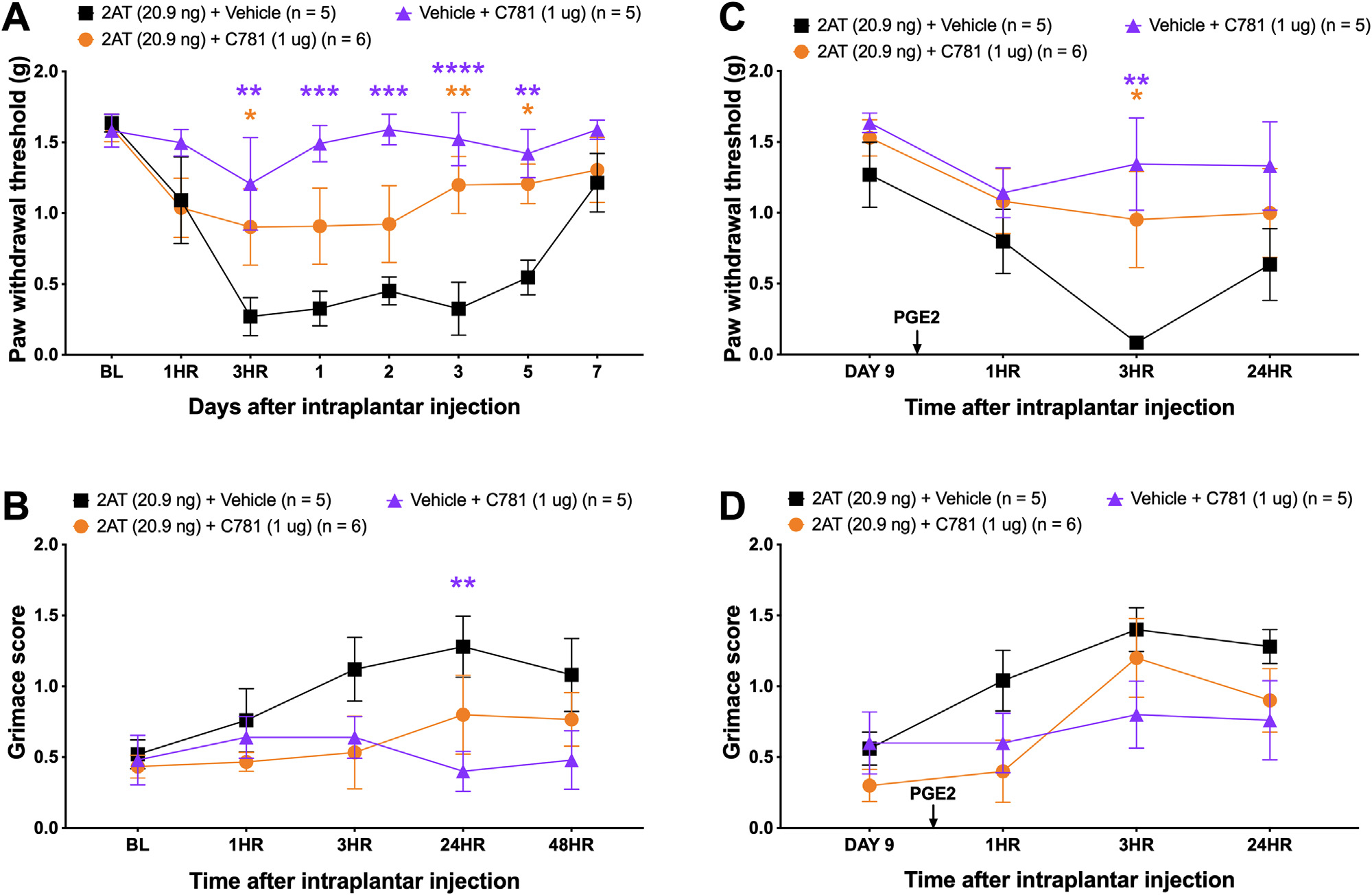
Local C781 decreases PAR2-mediated mechanical hypersensitivity and grimacing when coinjected with 2AT, a PAR2-specific agonist, and blocks hyperalgesic priming. Baseline (BL) measures were obtained before male and female C57BL6 mice received an intraplantar hind paw coinjection of 20.9 ng PAR2 agonist, 2AT, along with 1 *μ*g of PAR2 antagonist, C781. When compared to mice that received only 2AT, mice that received C781 showed decreased (A) acute mechanical sensitivity and (B) facial grimacing. Mice that received only C781 intraplantar injection showed no mechanical hypersensitivity nor grimacing, indicating that local C781 does not cause pain on its own. To assess for hyperalgesic priming, mice were allowed to return to baseline levels and then injected with 100 ng of PGE_2_ into the hind paw. (C and D) Mice that received C781 cotreatment showed decreased 2AT-induced hyperalgesic priming revealed by the loss of PGE_2_ response. For the 2AT + Vehicle group, n = 2 females and n = 3 males. For the 2AT + C781 group, n = 3 females and n = 3 males. For the Vehicle + C781 group, n = 2 females and n = 3 males. *P < .05, **P < .01, ***P < .001, ****P < .0001. For paw withdrawal threshold and grimace scores, repeated measures two-way ANOVA with Dunnett post-test was used. All group mean comparisons were made to the mean of the 2AT + Vehicle group.

We tested whether local C781 could block the effects of 2AT in simultaneous injection experiments. To do this we combined C781 and 2AT within 0.9% saline so that the final concentration was 194 *μ*M (100 *μ*g/mL) for C781 and 3 *μ*M (2.09 *μ*g/mL) for 2AT. The experimental group received 10 *μ*L volume of the combined dose of 1.94 nmols (1.0 *μ*g) C781 and 30 pmols (20.9 ng) 2AT, while the control groups received either 2AT (20.9 ng) or C781 (1.0 *μ*g) alone into the hind paw. 2AT caused both mechanical hypersensitivity and facial grimacing in mice. However, mice that received a coinjection of 2AT and C781 showed significantly less mechanical sensitivity, while grimace scores were not significantly changed (Fig 1A and 1B). Mice that received only C781 showed neither mechanical hypersensitivity nor facial grimacing. Once mice returned to baseline levels, we tested for hyperalgesic priming by injecting a subthreshold dose of PGE2 (100 ng intraplantar) and reassessed mechanical sensitivity and facial grimacing (Fig 1C and D). As expected, mice that were treated with 2AT primed and exhibited mechanical allodynia and facial grimacing in response to PGE2. Mice that received only C781 or C781 in conjunction with 2AT showed significantly less mechanical sensitivity 3 hours post-PGE2, while significant differences in facial grimacing were seen only in the C781 alone group. These results indicate that C781 does not intrinsically cause nociceptive responses and is able to block the sensitizing and priming effects of PAR2 activation via 2AT.

### Systemic C781 Dose-Dependently Attenuates PAR2-Evoked Nociception

**Figure 2.**
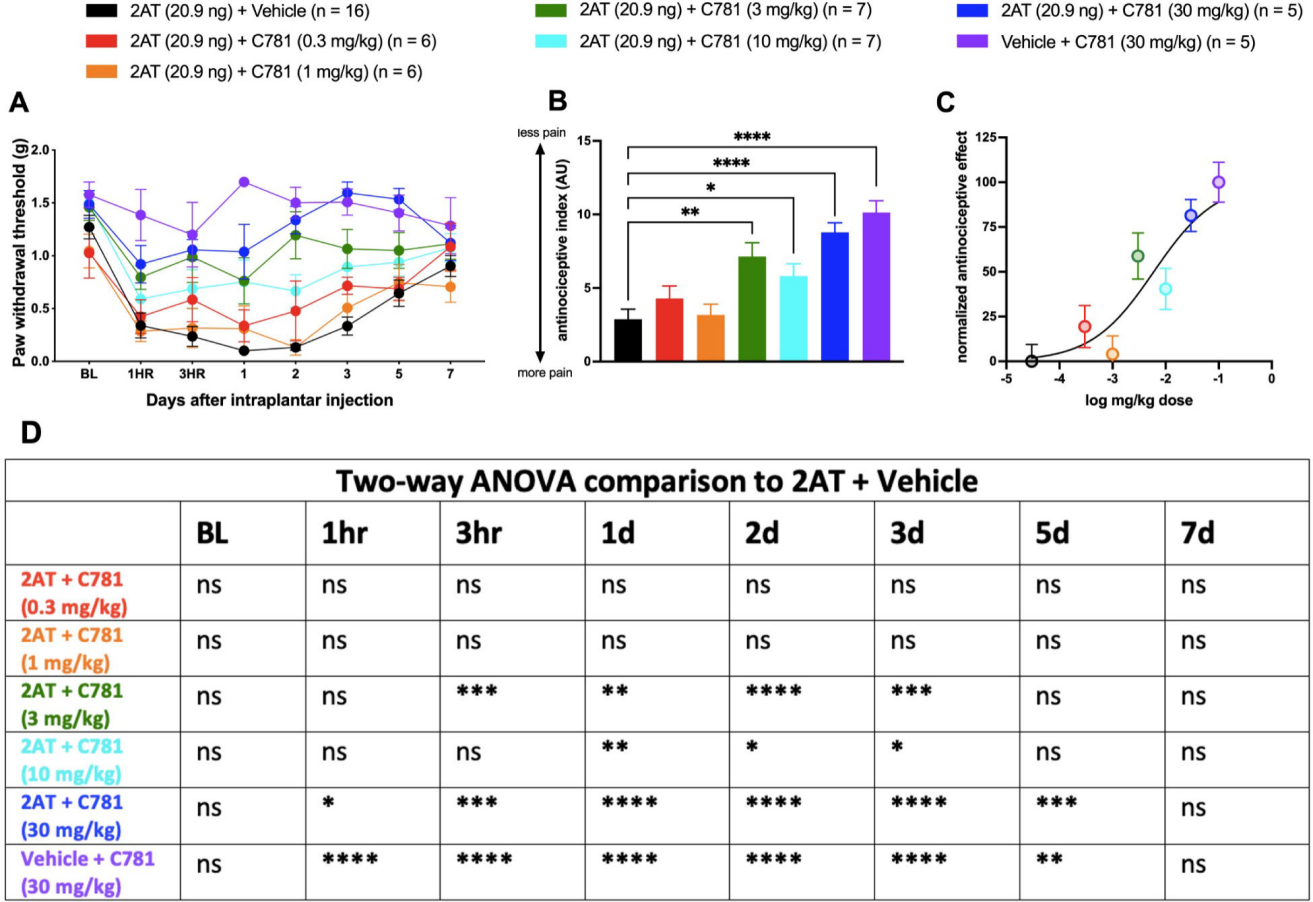
Dose-dependent effects of C781 systemic administration against 2AT-evoked mechanical hypersensitivity. Baseline (BL) paw withdrawal thresholds were obtained before male and female C57BL6 mice received an intraperitoneal injection of C781 or vehicle at the indicated doses, followed 30 minutes later by intraplantar hind paw injection of 2AT (20.9 ng). (A) von Frey paw withdrawal thresholds indicate that when compared to mice that received only 2AT, mice that received C781 showed a dose-dependent, decreased acute mechanical sensitivity. Mice that received only C781 intraperitoneal injection showed no mechanical hypersensitivity, indicating that systemic C781 does not cause pain on its own. (B) Antinociceptive index values were calculated for von Frey paw withdrawal thresholds for each group by calculating the area under curve from experiments in (A). Lower antinociceptive index indicates an overall lower paw withdrawal threshold and higher pain. Likewise, a higher antinociceptive index indicates an overall higher paw withdrawal threshold and lower pain. (C) A normalized dose-response curve of C781’s antinociceptive effect was obtained using the antinociceptive index values of each dosage. Based on this dose-response curve, we determined that the ED_50_ for intraperitoneal C781 to be 6.3 mg/kg for inhibition of PAR2 agonist-evoked pain. (D) Table with 2-way ANOVA comparisons of each dosage with the 2AT + Vehicle group. For the 2AT + Vehicle group, n = 8 females and n = 8 males. For the 2AT + C781 (0.3 mg/kg) group, n = 3 females and n = 3 males. For the 2AT + C781 (1 mg/kg) group, n = 3 females and n = 3 males. For the 2AT + C781 (3 mg/kg) group, n = 4 females and n = 3 males. For the 2AT + C781 (10 mg/kg) group, n = 4 females and n = 3 males. For the 2AT + C782 (30 mg/kg) group, n = 2 females and n = 3 males. For the Vehicle + C781 (30 mg/kg) group, n = 3 females and n = 2 males. *P < .05, **P < .01, ***P < .001, ****P < .0001. For antinociceptive index comparisons, 1-way ANOVA with Dunnett post-test was used, with all group comparisons made to the 2AT + Vehicle group.

We next sought to understand whether C781 can block PAR2-evoked nociception via systemic administration. We conducted a dose-response experiment using the following doses of C781: 0.3 mg/kg, 1 mg/kg, 3 mg/kg, 10 mg/kg, and 30 mg/kg. Mice were injected with C781 into the i.p. space an hour before intraplantar injection of 2AT (20.9 ng). Paw withdrawal thresholds were measured (Fig 2A), and the Area Under the Curves (AUCs) were then used to find the antinociceptive index for each dose (Fig 2B) and to graph the normalized antinociceptive effect (Fig 2C). Based on the dose-response curve, we determined that the ED_50_ for C781 was 6.3 mg/kg. We decided to use the 10 mg/kg dose as well as the higher 30 mg/kg dose for subsequent behavioral experiments. Animals that received only C781 with vehicle injections into the paw displayed no mechanical hypersensitivity, indicating that C781 on its own does not cause sensitization with systemic administration.

### Pharmacokinetic Properties of C781

**Figure 3.**
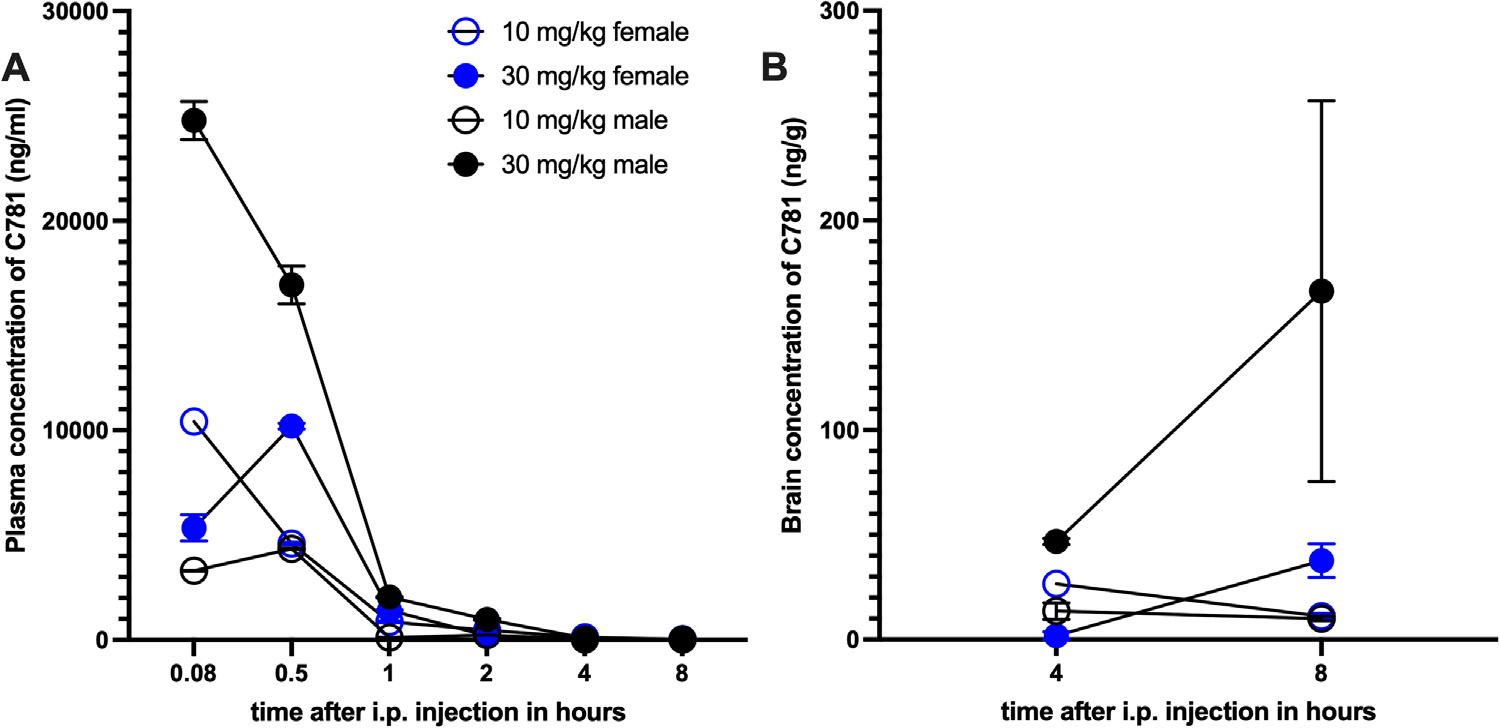
Plasma and brain C781 concentrations after a single intraperitoneal injection (10 and 30 mg/kg) in female and male C57BL6 mice. (A) Plasma concentrations of C781 after an i.p. injection of 10 or 30 mg/kg C781 in female and male mice. (B) Brain concentrations of C781 at 4 and 8 hours after i.p. dosing in female and male mice. N = 6 mice per dose, 3 of each sex.

We measured plasma and brain concentrations of C781 after i.p. dosing in male and female mice. In female mice, the 10 mg/kg dose had a time to maximal plasma concentration (T_max_) of 0.08 hours and a maximal plasma concentration (C_max_) of 10,418 ng/mL; the 30 mg/kg dose had a T_max_ of 0.5 hours and a C_max_ of 10,200 ng/mL (Fig 3A, Supplementary Document 1). In male mice, the 10 mg/kg dose had a T_max_ of 0.50 hours and a C_max_ of 4,350 ng/mL; the 30 mg/kg dose had a T_max_ of 0.08 hours and a C_max_ of 24,785 ng/mL (Fig 3A, Supplementary Document 2). We also measured brain concentrations of C781 at 4 and 8 hours after dosing and did not find evidence of appreciable brain accumulation of drug at these time points (Fig 3B). In females, the brain-to-plasma C781 ratio was 1.65 at the 30 mg/kg, 8 hour time point. In males, the brain-to-plasma C781 ratio was 8.42 at the 30 mg/kg, 8 hour time point. These data suggest a persistence of brain C781 levels with the higher peak plasma drug concentrations achieved in males at this dose.

We then measured C781 stability in mouse and human liver microsomes. Over the course of 60 minutes, C781 was not metabolized by mouse or human liver microsomes whereas the positive controls verapamil (human liver microsomes) and imipramine (mouse liver microsomes) were metabolized by more than 75% within 60 minutes (Supplementary Document 3). C781 showed moderate human and mouse plasma protein binding with 67.7 ± 2.6% and 60.7 ± 6.0%, respectively (Supplementary Document 4). Finally, we determined solubility of C781 at pH 2.0 (174.7 ± 2.8 mM) and pH 7.4 (162.6 ± 8.6 mM; Supplementary Document 5). These findings indicate the free drug concentration at the C_max_ point is estimated at 7.6 *μ*M in female mice and 19.2 *μ*M in male mice. Both of these concentrations are within the range where we observed PAR2 blockade in vitro.^54^ By 2 hours postdosing in both sexes, C781 free drug concentrations would be below 1 *μ*M where a substantial loss of receptor antagonism would be expected.

### C781 Both Prevents and Reverses Mechanical Hypersensitivity and Facial Grimacing Caused by Neutrophil Elastase, an Endogenous PAR2-Cleaving Enzyme

Having determined that C781 effectively blocks nociception caused by a PAR2 peptidomimetic agonist, our next goal was to test whether C781 can inhibit nociception induced by endogenous protease that act via PAR2. Neutrophil elastase, a serine protease released by neutrophils and macrophages at sites of inflammation, is a known biased agonist of PAR2.^50,64^ Instead of activating G_q_-coupled Ca^2+^ signaling, neutrophil elastase selectively activates the *β*-arrestin/MAPK pathway, a signaling pathway that our previous work showed is the underlying mechanism behind PAR2-mediated mechanical hypersensitivity, facial grimacing, and hyperalgesic priming.^57^

**Figure 4.**
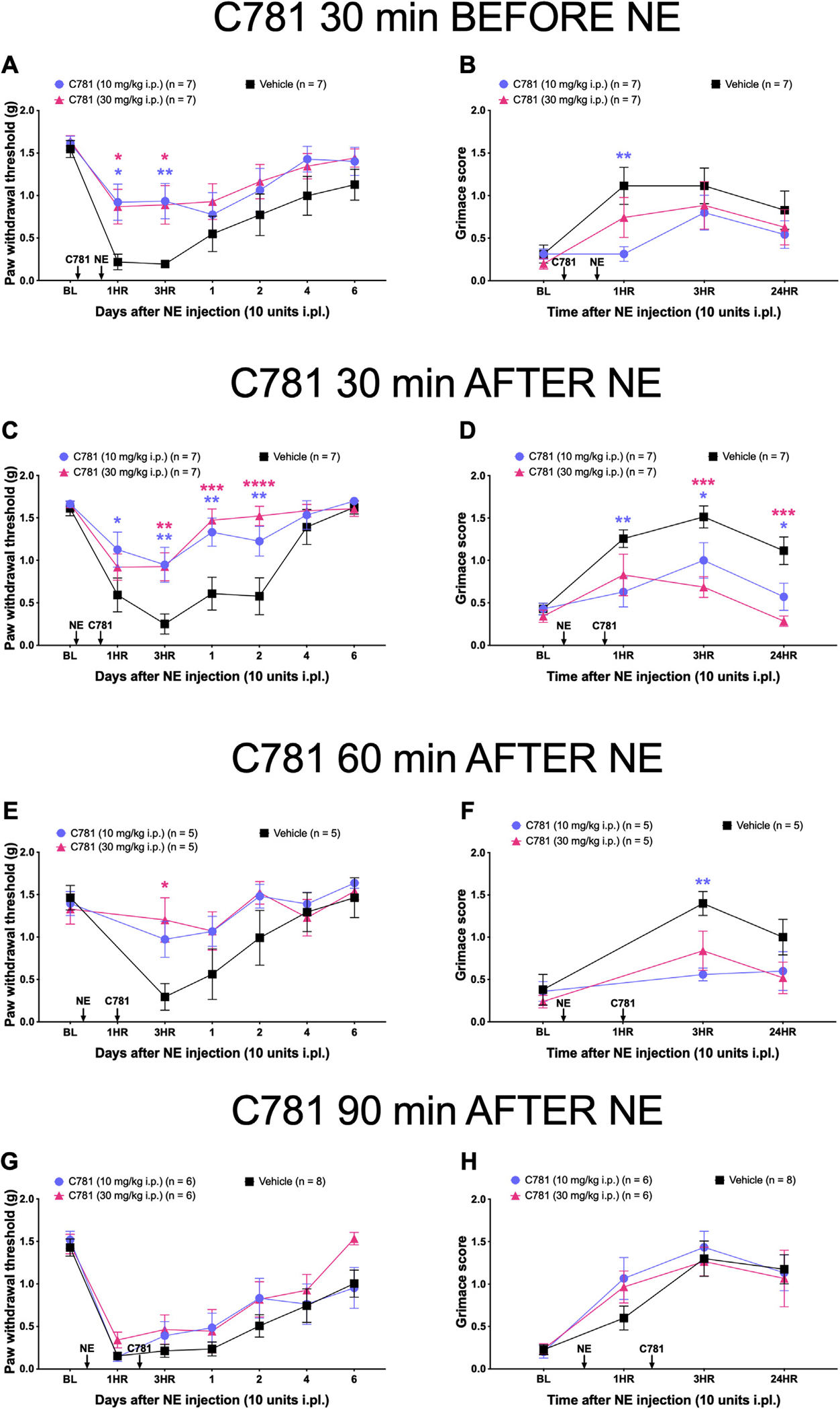
C781 blocks and also reverses the effects of neutrophil elastase, an endogenous PAR2-cleaving enzyme, up to 60 minutes poststimulus. Baseline (BL) paw withdrawal thresholds and grimace scores for male and female ICR mice were obtained before hind paw injection with neutrophil elastase (NE) and C781 intraperitoneal injection. C781 (10 and 30 mg/kg i.p.) was injected (A and B) 30 minutes before, (C and D) 30 minutes after, (E and F) 60 minutes after, and (G and H) 90 minutes after NE (10 units) paw injection, and (A, C, E, G) von Frey withdrawal thresholds and (B, D, F, H) grimace scores were each assessed after the NE injection. For the experiment injecting C781 60 minutes after NE, the 1 hour time point was skipped since it coincided with C781 injection time. In the “C781 30 minutes before NE” experiment, the C781 10 mg/kg group had n = 4 females and n = 3 males, the C781 30 mg/kg group had n = 3 females and n = 4 males, and the Vehicle group had n = 3 females and n = 4 males. In the “C781 30 minutes after NE” experiment, the C781 10 mg/kg group had n = 4 females and n = 3 males, the C781 30 mg/kg group had n = 3 females and n = 4 males, and the Vehicle group had n = 2 females and n = 5 males. In the “C781 60 minutes after NE” experiment, the C781 10 mg/kg group had n = 2 females and n = 3 males, the C781 30 mg/kg group had n = 2 females and n = 3 males, and the Vehicle group had n = 3 females and n = 2 males. In the “C781 90 minutes after NE” experiment, the C781 10 mg/kg group had n = 3 females and n = 3 males, the C781 30 mg/kg group had n = 3 females and n = 3 males, and the Vehicle group had n = 4 females and n = 4 males. *P < .05, **P < .01, ***P < .001, ****P < .0001. For paw withdrawal threshold and grimace scores, repeated measures 2-way ANOVA with Dunnett post-test was used. All group mean comparisons were made to the mean of the Vehicle group.

We injected C781 (10 and 30 mg/kg i.p.) 30 minutes before injecting neutrophil elastase (10 units intraplantar) (Fig 4A and 4B). Compared to vehicle-treated animals, the mice given C781 at both dosages showed significantly less mechanical hypersensitivity at 1 and 3 hour postneutrophil elastase. For facial grimace scores, 10 mg/kg C781 significantly attenuated grimacing at the 1 hour time point. These results show that C781 can prevent both mechanical hypersensitivity and facial grimacing caused by neutrophil elastase when given prior to protease treatment.

While this was a positive outcome, most pain medicines are taken after the onset of pain, so an important test of the potential of C781 as a therapeutic is in its ability to reverse PAR2-mediated nociception. To test this hypothesis, we first injected neutrophil elastase into the paw and then injected C781 i.p. at 30, 60, or 90 minutes after neutrophil elastase dosing. At 30 minutes after neutrophil elastase (Fig 4C and 4D), both 10 mg/kg and 30 mg/kg C781 significantly reduced mechanical hypersensitivity facial grimacing after neutrophil elastase when compared to the vehicle group. Compared to vehicle-treated animals, mice given 10 mg/kg C781 showed a significant difference in paw withdrawal threshold at the 1 hour, 3 hour, 1 day, and 2 day time point and at the 1 hour, 3 hour, and 24 hour time point for facial grimacing. For the mice given 30 mg/kg C781, they showed a significant difference to vehicle-treated animals in paw withdrawal threshold at the 3 hour, 1 day, and 2 day time point and at the 3 hour and 24 hour time point for facial grimacing. At 60 minutes after neutrophil elastase (Fig 4E and 4F), 30 mg/kg C781 significantly reduced mechanical hypersensitivity at the 3 hour time point. For these experiments, the 1 hour time point was not measured due to it coinciding with the C781 injection. Facial grimacing was also significantly reduced with 10 mg/kg C781 at the 3 hour time point. However, by the 90 minutes mark after neutrophil elastase (Fig 4G and 4H), C781-treated animals showed similar paw withdrawal thresholds and facial grimace scores as the vehicle-treated group. These experiments demonstrated that C781 can prevent neutrophil elastase-mediated mechanical hypersensitivity and facial grimacing and is also able to reverse these nociceptive behaviors when given up to and less than 60 minutes after neutrophil elastase.

### C781 Attenuates Mechanical Hypersensitivity and Facial Grimacing Caused by Compound 48/80, a Mast Cell Degranulator

**Figure 5.**
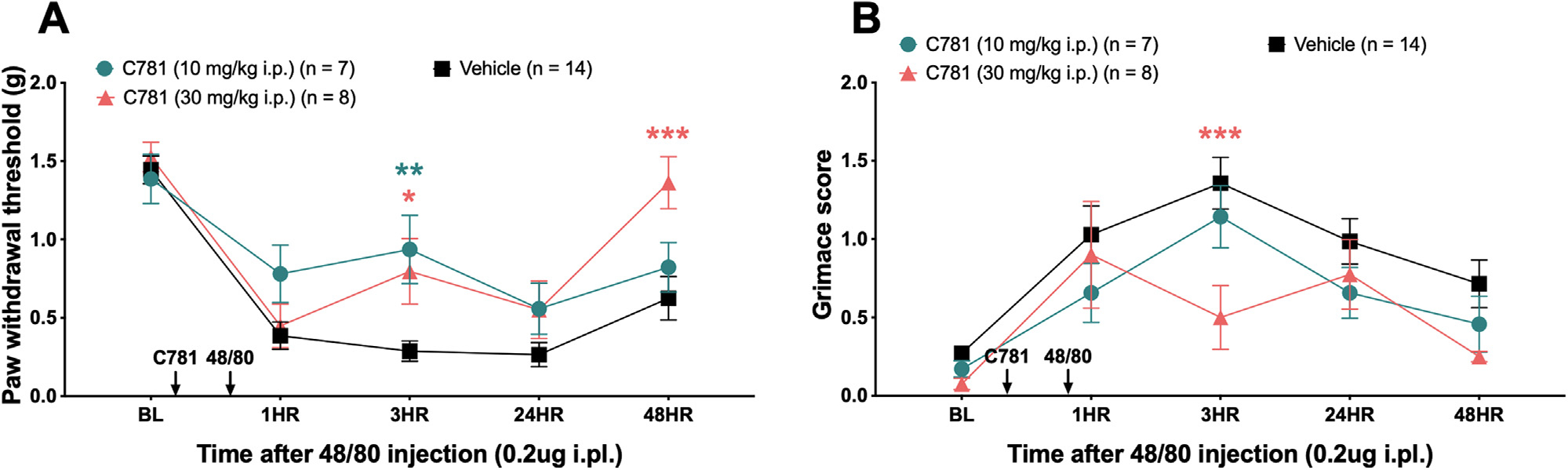
C781 attenuates the pronociceptive effects of the mast cell degranulator, compound 48/80. Baseline (BL) paw withdrawal thresholds and grimace scores for male and female ICR mice were obtained before hind paw injection with compound 48/80 and C781 intraperitoneal injection. C781 (10 and 30 mg/kg i.p.) was injected 30 minutes before compound 48/80 (0.2 ug i.pl.) paw injection, and (A) von Frey withdrawal threshold and (B) grimace scores were assessed after compound 48/80 injection. For the C781 (10 mg/kg) group, n = 4 females, and n = 3 males. For the C781 (30 mg/kg) group, n = 4 females and n = 4 males. For the Vehicle group, n = 6 females and n = 8 males. *P < .05, **P < .01, ***P < .001. For paw withdrawal threshold and grimace scores, repeated measures 2-way ANOVA with Dunnett post-test was used. All group mean comparisons were made to the mean of the Vehicle group.

Another potential source of endogenous PAR2-activating proteases are mast cells. These immune cells release a wide range of enzymes and inflammatory factors, including mast cell tryptase, and have been proposed to be an important mediator for migraine headaches by acting on PAR2 expressed on meningeal nociceptors.^22,25,63^ To activate mast cells, we injected compound 48/80 into the paw 30 minutes after C781 and measured paw withdrawal thresholds and grimace scores (Fig. 5A and 5B). Intraplantar injection of compound 48/80 caused mechanical hypersensitivity and facial grimacing in the vehicle treatment group. C781 inhibited the mechanical hypersensitivity at the 3 hour time point (10 and 30 mg/kg) and 48 hour time point (30 mg/kg), as well as facial grimacing at the 3 hour time point (30 mg/kg). Given that the degranulated mast cells release more than just tryptase,^17^ non-PAR2-activating inflammatory factors likely also contribute to the mechanical sensitivity and facial grimacing observed in these experiments.

### C781 Does not Prevent Mechanical Hypersensitivity and Facial Grimacing Caused by λ-Carrageenan, a Nonspecific Inflammatory Agent

**Figure 6.**
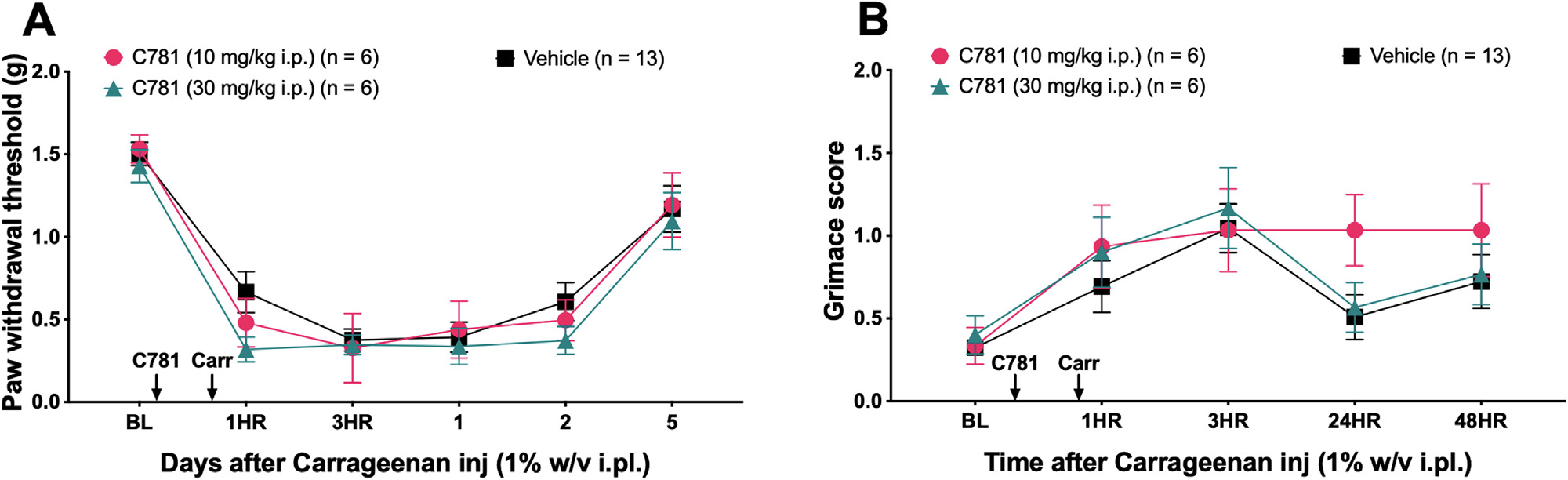
C781 does not block the nociceptive effects of the inflammatory agent *λ*-carrageenan. Baseline (BL) paw withdrawal thresholds and grimace scores for male and female ICR mice were obtained before hind paw injection with *λ*-carrageenan and C781 intraperitoneal injection. C781 (10 and 30 mg/kg i.p.) was injected 30 minutes before *λ*-carrageenan (1% w/v) paw injection, and (A) von Frey withdrawal threshold and (B) grimace scores were assessed after carrageenan injection. For the C781 (10 mg/kg) group, n = 3 females and n = 3 males. For the C781 (30 mg/kg) group, n = 2 females and n = 4 males. For the Vehicle group, n = 6 females and n = 7 males. For paw withdrawal threshold and grimace scores, repeated measures 2-way ANOVA with Geisser-Greenhouse correction with Dunnett post-test was used. All group mean comparisons were made to the mean of the Vehicle group.

We next tested C781 against a nonspecific inflammatory stimulus, *λ*-carrageenan. *λ*-Carrageenan causes acute inflammation via COX-2 and the release of histamine, serotonin, prostaglandins, proteases, and lysosomes.^16,47^ While these are not traditional PAR2 activators, *λ*-carrageenan responses have been shown to be decreased in PAR2 knockout mice.^61^ Taken together, it is not clear if this general inflammatory hyperalgesic response would be blocked by a biased antagonist like C781. To test this, we injected *λ*-carrageenan (1% w/v) into the hind paw of mice 30 minutes after C781 (10 and 30 mg/kg i.p.) (Fig 6A and 6B). C781 had no effect on mechanical hypersensitivity or facial grimacing caused by *λ*-carrageenan injection.

## Discussion

In this report we describe the in vivo pharmacology of a novel, biased PAR2 antagonist, C781. Our findings demonstrate that C781 can act locally or systemically and is effective at blocking mechanical hypersensitivity and facial grimacing, a measure of affective pain, in response to a PAR2 agonist, neutrophil elastase, or mast cell mediators. Our findings suggest that protease-driven nociceptive behaviors are mediated by *β*-arrestin/MAPK activation downstream of PAR2 because this is the signaling pathway blocked by C781. PK measurements paralleled PD responses in vivo, consistent with a specific action of the biased antagonist in mice. C781 has good systemic bioavailability but future compounds in this series should focus on increasing the plasma half-life. Carrageenan-evoked pain has been linked to PAR2 activation using knockout mice,^61^ but C781 had no effect in this model suggesting roles of other receptors, or nonprotease mediated actions in the nociceptive behaviors caused by carrageenan. Another possibility is that the proteases involved in carrageenan-evoked nociception require Ca^2+^ signaling downstream of PAR2 but not *β*-arrestin/MAPK activation. We conclude that C781 exemplifies a path forward for development of PAR2 antagonists to interrupt pain driven by proteases in humans. A priority will be optimization of PK properties in future C781-like molecules.

With respect to the PK of C781, we noted substantial differences between male and female animals, in particular at early time points. The mechanisms underlying these differences are not know but could involve sex differences in transporter expression.^18,23^ The drug was given i.p. in all animals so transporter-mediated absorption could contribute to sex differences in drug plasma levels, in particular, at early time points. Understanding these sex differences at the molecular level is outside the scope of this study for 2 reasons. The first is that the free drug plasma concentrations for both doses (10 and 30 mg/kg) in males and females at the 30 minutes post-dose time point are above 1 *μ*M. Based on our in vitro pharmacology reported in our previous work,^54^ we would expect block of PAR2 signaling via *β*-arrestin/MAPK at this concentration. These effects were born out in the behavioral findings, giving a good match between PK and in vivo pharmacodynamics in both sexes. The second reason is that we intend to do PK optimization for C781 for oral dosing and we do not know if the PK differences would be similar with that dosing route. As noted above, a major goal of future work will be to increase the plasma half-life after oral dosing for C781-like compounds while maintaining potency and efficacy at human PAR2.

For 2 decades it has been known that mice lacking PAR2 display a decrease in pain behaviors following exposure to many endogenous proteases, including mast cell proteases, but several factors have hindered translation of these widely replicated findings into a clinically tested drug.^42,51,61^ It has proven difficult to develop specific ligands to probe PAR2 function in vivo. An example is that the widely used SLIGRL PAR2 activating peptide is nonspecific and binds to Mrgprs (Mas-related G protein-coupled receptors),^39^ complicating the interpretation of experiments done with this tool. It was later discovered that certain PAR2-cleaving proteases, like cathepsin S, can also activate Mrgprs,^52^ further complicating interpretation of physiological roles of PAR2 in sensory diseases like itch and pain. The development of antagonists has accelerated in recent years. There are now function blocking antibodies,^3,33^ PAR2 pepducins that prevent G-protein coupling,^55^ negative allosteric modulators ^13^ and Ca^2+^-biased antagonists to PAR2.^62^ C781 is the first *β*-arrestin/MAPK-biased antagonist of PAR2.^54^ Given the key role that this signaling pathway plays in pain responses and nociceptor sensitization,^48,57^ we propose that this type of antagonist is well-suited for further development for pain treatment. We have not directly assessed the selectivity of C781 for PAR2 versus other GPCRs in the PAR family, or other GPCRs. This will be a focus as we continue to develop this series of compounds with optimized pharmacokinetics.

The question, then, is what type of pain? Our findings show that C781 was effective for protease-evoked nociceptive behaviors but not for the general inflammatory mediator carrageenan. Our interpretation of this finding is that further development should be aimed at diseases where pain can be linked to increases in specific proteases. An example may be subpopulations of osteoarthritis patients where neutrophil elastase is increased in joints. Previous studies have shown neutrophil elastase increases in mouse osteoarthritis models. PAR2 antagonists and PAR2 knockout reduces nociceptive behaviors in these mice.^45^ These nociceptive behaviors are also reversed by MAPK inhibition. Future experiments with C781 or similar compounds can directly assess whether this PAR2-mediated signaling is important for nociceptive behaviors in these models in rodents. Given that neutrophil elastase produces a long-lasting behavioral effect and acts via MAPK signaling, this serine protease is an important target for identification of patient populations that may benefit from PAR2 biased antagonism. Because proteases can be measured in samples from patients, for instance from synovial fluid, this creates a rationale for a personalized or precision medicine approach where patients are selected for PAR2 antagonist treatment based on a protease profile. Our view is that this path can lead to the highest chances of clinical success.

PAR2 is expressed in many tissues outside of the peripheral nervous system, including the respiratory tract, gastrointestinal tract, central nervous system and cardiovascular system, with important physiological and pathophysiological functions.^9,14^ While such broad expression of PAR2 certainly highlights the potential for PAR2-targeted therapeutics to treat a wide range of diseases, it also creates the concern for adverse effects with PAR2 blocking approaches. One way to avoid this issue is to engineer antagonists of PAR2 that block certain signaling functions but leave others intact. Consistent with this notion, PAR2 is known to behave in a biased manner through multiple signaling transducers, with distinct biochemical and physiological responses in different tissue types.^28,56^ An important example of this is the endogenous protease neutrophil elastase, which cleaves the entire extracellular N-terminus of PAR2 and selectively induces MAPK signaling to promote physiological responses including pain.^45,50^ Another example is legumain, which was recently identified as an important mediator of cancer pain, signaling through PAR2 in a biased fashion via Ca^2+^ and cyclic AMP.^58^ Pharmacologic targeting of select signaling pathways activated by PAR2 may prove beneficial without affecting important physiological functions of PAR2. In the context of pain, we have shown that the MAPK/ERK pathway mediates mechanical hypersensitivity and spontaneous nociceptive behaviors from PAR2 activation with peptidomimetic agonists derived from the trypsin-cleaved sequence.^57^ The biased antagonist approach will likely require a thorough understanding of the physiological “activator” of PAR2 in the disease state so that the appropriate signaling pathway can be targeted. As detailed above, certain models of osteoarthritis may offer an excellent way to test this in a translationally-relevant way.^45^

Using the specific PAR2 agonist, 2AT, we found that a local coinjection of C781 attenuated both the acute pain response and hyperalgesic priming, consistent with blockade of MAPK signaling downstream of PAR2 in vivo.^57^ With systemic dosing of C781, again using 2AT as the PAR2 activating stimulus, we determined the C781 ED_50_ to be 6.3 mg/kg. Taking into account the PK data, this indicates that free drug concentrations of 1 *μ*M or greater are needed to inhibit 2AT-evoked pain in vivo. This finding aligns with the in vitro pharmacology described in our published description of C781.^54^ An important insight from our work is that C781 not only blocks mechanical hypersensitivity and facial grimacing from neutrophil elastase, but that it can also reverse the nociception sensitizing effects of neutrophil elastase for up to 60 minutes postprotease exposure. PAR2 activation by proteolytic cleavage is an irreversible process that leads to receptor signaling at the membrane and at signaling endosomes following receptor internalization.^30,34^ Our interpretation of the reversal effect is that C781 interferes with plasma membrane and endosomal PAR2 signaling and this can interrupt pain promoting effects of neutrophil elastase caused by signaling from this compartment.^30^ This would likely be an important feature of PAR2-targeting therapeutics for pain as the medication would likely be taken following engagement of protease signaling that promotes the pain state. We hypothesize that our PAR2 biased antagonist described here can be modified to improve PK properties (longer in vivo plasma half-life) that would then increase in vivo efficacy. Optimizing PK while maintaining favorable PD properties will be a focus for future work.

## Supplementary Material

1

2

3

4

5
