# Supplementary material for: C781, a β-Arrestin Biased Antagonist at Protease-Activated Receptor-2 (PAR2), Displays in vivo Efficacy Against Protease-Induced Pain in Mice
Source: J Pain. Author manuscript; Available in PMC 2023 Apr 7. (PMC10079573; doi:10.1016/j.jpain.2022.11.006)
Supplement: 1 [file NIHMS1851843-supplement-1.pdf]

Sai Life Sciences Limited

PHARMACOKINETIC REPORT

Study Number: SAIDMPK/PK-19-10-849

Study Title

Plasma pharmacokinetics and tissue (brain) distribution of Compound-781 following single intraperitoneal (Dose: 10 and 30 mg/kg) dose administration in female C57BL/6 mice

Sponsor

Theodore Price PhD
University of Texas at Dallas
BSB 14.102G 800 W Campbell Rd
Richardson
TX 75080 phone: 972-883-4311
cell: 520-471-0360 fax: 972-883-2491
theodore.price@utdallas.edu

Test Facility

DMPK, Sai Life Sciences Limited
Building 1, Plot 2, Chrysalis Enclave
International Biotech Park, Phase II Hinjewadi
Pune - 411 057 INDIA
Phone: +91-20-30125000

Date of Completion of Report: November 2019

[redacted]

[redacted]

Contents

List of Tables 3
List of Figures 4
List of Abbreviations 5
Study Responsibilities 6
1.0 Summary 7
2.0 Study Objective 9
3.0 Test Guidelines / SOPs / Compliance 9
4.0 Animal Welfare 9
5.0 Experimental 9
5. 1 Test Compound 9
5. 2 Test System 9
5. 3 Study Design 10
5. 4 Formulation Preparation 10
5.4.1 Formulation Results 11
5. 5 Observations 11
5. 6 Sample Collection 11
5. 7 Bioanalysis 11
6.0 Data Analysis 12
7.0 Results 12
8.0 Data Archiving 12
9.0 Annexure I 17

List of Tables

Table 1: Plasma pharmacokinetic parameters of Compound-781 following a single intraperitoneal dose administration to female C57BL/6 mice (Dose: 10 and 30 mg/kg) .. 13
Table 2: Individual plasma concentration-time data of Compound-781 following a single intraperitoneal administration to female C57BL/6 mice (Dose: 10 mg/kg) 14
Table 3: Individual plasma concentration-time data of Compound-781 following a single intraperitoneal administration to female C57BL/6 mice (Dose: 30 mg/kg) 14
Table 4: Mean brain-to-plasma concentration ratio of Compound-781 following a single intraperitoneal administration to female C57BL/6 mice (Dose: 10 and 30 mg/kg) 15

List of Figures

Figure 1: Mean plasma concentration-time profiles of Compound-781 following a single intraperitoneal dose administration to female C57BL/6 mice (Dose: 10 and 30 mg/kg) ..16

List of Abbreviations

**LLOQ**	Lower Limit of Quantitation
**CPCSEA**	Committee for the Purpose of Control and Supervision of Experiments on Animals
**CV**	Coefficient of Variation
**IAEC**	Institutional Animal Ethics Committee
**IS**	Internal Standard
**LC-MS/MS**	Liquid Chromatography Mass Spectrometry
**NA**	Not Applicable
**IP**	Intraperitoneal
**SD**	Standard Deviation
**SOP**	Standard Operating Procedure
C_{max}	Maximum concentration
T_{max}	Time to reach maximum concentration
**AUC**	Area under Plasma concentration - Time curve

Study Responsibilities

**Study Director :**	Aslam Burhan, Ph.D.
**Formulation:**	Anandkumar Yadav, M. Pharm.
**In-Life Phase:**	Mahesh Rahinj, M. Pharm.<br>Amol S. Rasal, M. Pharm.<br>Nilkanth Naik, M. Pharm.<br>Anandkumar Yadav, M. Pharm.<br>Suraj Lanke, M. Pharm.<br>Sanjay Bedare, M. S.
**Bioanalysis:**	Srikant Jadhav, M. Pharm.
**Data Analysis and Report Prepared By:**	Nilkanth Naik, M. Pharm.
**QC By:**	Sapana Chavan, M.Sc.
**Report Reviewed By:**	Aslam Burhan, Ph.D.

1.0 Summary

The objective of this study was to investigate the plasma pharmacokinetics and brain distribution of Compound-781 following single intraperitoneal dose administration in female C57BL/6 mice at 10 and 30 mg/kg dose. A group of twelve female mice were divided into two groups as:

Group 1; 10 mg/kg/IP; (n = 6; Animal # 1-6),

Group 2; 30 mg/kg/IP; (n = 6; Animal # 7-12).

Animals in Group 1 and Group 2 were administered intraperitoneally with Compound-781 solution formulation in normal saline at 10 and 30 mg/kg dose respectively.

Blood samples (approximately 60 μL) were collected under light isoflurane anesthesia from retro orbital plexus such that samples were obtained at 0.08, 0.5, 1, 2, 4 and 8 hr (IP). At each time point blood samples were collected from three mice. Immediately after collection, plasma was harvested by centrifugation and stored at -70ºC until analysis..

Immeadiately after collection of blood, animals were euthanized with excess CO_2 asphyxiation. Brain samples were collected from mice at 4 and 8 hr from respective animal. Brain samples were homogenized using ice-cold phosphate buffer saline (pH-7.4). Total homogenate volume was three times the brain weight.

All samples were processed for analysis by protein precipitation using acetonitrile and analyzed with fit-for-purpose LC/MS/MS method (LLOQ = 1.01 ng/mL for plasma, 3.03 ng/g for brain).

Pharmacokinetic parameters were calculated using the non-compartmental analysis tool of Phoenix WinNonlin (Version 7.0). The overall pharmacokinetic parameters are summarized below:

Matrix	Route	Dose (mg/kg)	T_{max} (hr)	C_{max} (ng/mL)	AUC_{last} (hr*ng/mL)	AUC_{inf} (hr*ng/mL)
Plasma	IP	10	0.08	10417.78	6472.28	6544.75
		30	0.50	10199.77	7605.39	7668.28

10 mg/kg/IP: Following a single intraperitoneal dose administration of Compound-781 at 10 mg/kg to female C57BL/6 mice, the plasma concentrations were observed up to 8 hr with T_{max} at 0.08 hr. Brain concentrations were quantifiable at 4 and 8 hr, Brain-to-plasma ratio were 0.23 (4 hr) and 0.34 (8 hr).

30 mg/kg/IP: Following a single intraperitoneal dose administration of Compound-781 at 30 mg/kg to female C57BL/6 mice, the plasma concentrations were observed up to 8 hr with T_{max} at 0.5 hr. Brain concentrations were quantifiable at 4 and 8 hr, Brain-to-plasma ratio were 0.09 (4 hr) and 1.65 (8 hr).

2.0 Study Objective

To investigate the plasma pharmacokinetics and brain distribution of Compound-781 following single intraperitoneal dose administration in female C57BL/6 mice at 10 and 30 mg/kg dose..

3.0 Test Guidelines / SOPs / Compliance

The study was conducted at Sai Life Sciences Limited, Pune, India, in accordance with the Study Plan SAIDMPK/PK-19-10-849.

This study was performed with approval of Institutional Animal Ethics Committee (IAEC) in accordance with requirement of The Committee for the Purpose of Control and Supervision of Experiments on Animals (CPCSEA), India. The study was not performed as per GLP regulations and not audited by QA; however all appropriate documentation is maintained in study file. Study phases, data generated and the report have been verified for the accuracy by the study group.

4.0 Animal Welfare

All procedures of the present study were in accordance with the guidelines provided by the Committee for the Purpose of Control and Supervision of Experiments on Animals (CPCSEA) as published in The Gazette of India, December 15, 1998. Prior approval of the Institutional Animal Ethics Committee (IAEC) was obtained before initiation of the study.

5.0 Experimental

5. 1 Test Compound

The test compound Compound-781 (Mol. Wt: 515.30, Purity: 98.142%) was received from Sponsor.

5. 2 Test System

Healthy female C57BL/6 mice (8-12 weeks old) weighing between 20 to 35 g were procured from ACTREC, India. Three mice were housed in each cage. Temperature and humidity were maintained at 22 ± 3 ºC and 30-70%, respectively and illumination was controlled to give a sequence of 12 hr light and 12 hr dark cycle. Temperature and humidity were recorded by auto-controlled data logger system. All

the animals were provided laboratory rodent diet (Envigo Research private Ltd, Hyderabad). Reverse osmosis water treated with ultraviolet light was provided *ad libitum*.

5. 3 Study Design

A group of twelve female mice were divided into two groups as:

Group 1; 10 mg/kg/IP; (n = 6; Animal # 1-6),

Group 2; 30 mg/kg/IP; (n = 6; Animal # 7-12).

Animals in Group 1 and Group 2 were administered intraperitoneally with Compound-781 solution formulation in normal saline at 10 and 30 mg/kg dose respectively.

The dosing volume administered was 10 mL/kg. The assignment of animals was shown in the table below:

Group	Route	Dose (mg/kg)	Animal ID
1	IP	10	6 (1- 6)
2	IP	30	6 (7-12)

5. 4 Formulation Preparation

The strengths of intraperitoneal solution formulations were 1 mg/mL and 3 mg/mL.

Ingredients	IP (1 mg/mL)	IP (3 mg/mL)
Compound-781	2.30 mg	6.73 mg
Normal saline (100%)	2.257 mL	2.201 mL

1 mg/mL: Accurately weighed quantity 2.30 mg of Compound-781 for IP dosing was added in a labeled bottle. The volume 2.257 mL of normal saline was added. The formulation was vortexed for 2 minutes to get clear solution.

3 mg/mL: Accurately weighed quantity 6.73 mg of Compound-781 for IP dosing was added in a labeled bottle. The volume 2.201 mL of normal saline was added. The formulation was vortexed for 2 minutes to get clear solution.

5.4.1 Formulation Results

After preparation of formulations, a volume of 200 µL was aliquoted for analysis. The formulations were analyzed and found to be within the acceptance criteria (in-house acceptance criteria is ± 20% from the nominal value). Formulations were prepared freshly prior to dosing.

Compound	Formulation	Theoretical Conc. (mg/mL)	Conc. Found (mg/mL)	% Change
Compound-781	IP	1.00	0.99	-1.00
		3.00	2.97	-1.00

5. 5 Observations

All the animals were found to be normal without showing any clinical signs after intraperitoneal dose administration at 10 and 30 mg/kg dose.

5. 6 Sample Collection

Blood: Blood samples (approximately 60 µL) were collected under light isoflurane anesthesia from retro orbital plexus such that samples were obtained at 0.08, 0.5, 1, 2, 4 and 8 hr (IP). At each time point blood samples were collected from three mice. Immediately after collection, plasma was harvested by centrifugation and stored at -70ºC until analysis..

Brain: Immeadiately after collection of blood, animals were euthanized with excess CO_2 asphyxiation. Brain samples were collected from mice at 4 and 8 hr from respective animal. Brain samples were homogenized using ice-cold phosphate buffer saline (pH-7.4). Total homogenate volume was three times the brain weight.

5. 7 Bioanalysis

Concentrations of Compound-781 in mouse plasma and brain samples were determined by fit for purpose LC-MS/MS method. The sample processing and extraction procedure, chromatographic and mass spectrometric conditions were presented in Annexure I.

6.0 Data Analysis

Non-compartmental analysis module in Phoenix WinNonlin® (Version 7.0) was used to assess the pharmacokinetic parameters. Maximum concentration (C_{max}) and time to reach maximum concentration (T_{max}) were the observed values. The areas under the concentration time curve (AUC_{last} and AUC_{inf}) and elimination half-life was calculated by linear trapezoidal rule. The terminal elimination rate constant, ke was determined by regression analysis of the linear terminal portion of the log plasma concentration-time curve.

7.0 Results

10 mg/kg/IP: Following a single intraperitoneal dose administration of Compound-781 at 10 mg/kg to female C57BL/6 mice, the plasma concentrations were observed up to 8 hr with T_{max} at 0.08 hr. Brain concentrations were quantifiable at 4 and 8 hr, Brain-to-plasma ratio were 0.23 (4 hr) and 0.34 (8 hr).

30 mg/kg/IP: Following a single intraperitoneal dose administration of Compound-781 at 30 mg/kg to female C57BL/6 mice, the plasma concentrations were observed up to 8 hr with T_{max} at 0.5 hr. Brain concentrations were quantifiable at 4 and 8 hr, Brain-to-plasma ratio were 0.09 (4 hr) and 1.65 (8 hr).

8.0 Data Archiving

All raw data, study protocol, and final report were documented and will be archived. The materials (hard and soft copies) will be retained for 1 year from the date of approval of final report. Thereafter, the archived material will be destroyed or stored for extended period as per written consent from the sponsor.

Table 1: Plasma pharmacokinetic parameters of Compound-781 following a single intraperitoneal dose administration to female C57BL/6 mice (Dose: 10 and 30 mg/kg)

Matrix	Route	Dose (mg/kg)	T_{max} (hr)	C_{max} (ng/mL)	AUC_{last} (hr*ng/mL)	AUC_{inf} (hr*ng/mL)
Plasma	IP	10	0.08	10417.78	6472.28	6544.75
		30	0.50	10199.77	7605.39	7668.28

Table 2: Individual plasma concentration-time data of Compound-781 following a single intraperitoneal administration to female C57BL/6 mice (Dose: 10 mg/kg)

Animal ID	Plasma concentration (ng/mL)					
	Time (hr)					
	**0.08**	**0.5**	**1**	**2**	**4**	**8**
1	10589.02		794.46		114.67	
2	9856.35		872.61		110.18	
3	10807.96		925.12		116.76	
4		4501.08		451.49		33.94
5		4606.27		495.97		29.20
6		4695.43		440.65		38.41
**Mean**	**10417.78**	**4600.93**	**864.06**	**462.70**	**113.87**	**33.85**
SD	498.38	97.29	65.75	29.32	3.36	4.61
CV%	4.78	2.11	7.61	6.34	2.95	13.61

LLOQ = 1.01 ng/mL

Table 3: Individual plasma concentration-time data of Compound-781 following a single intraperitoneal administration to female C57BL/6 mice (Dose: 30 mg/kg)

Animal ID	Plasma concentration (ng/mL)					
	Time (hr)					
	**0.08**	**0.5**	**1**	**2**	**4**	**8**
7	5498.47		1448.07		61.97	
8	6332.45		1432.99		61.15	
9	4200.02		1363.87		58.90	
10		10008.26		213.05		22.95
11		10120.41		189.31		21.80
12		10470.64		173.31		24.99
**Mean**	**5343.65**	**10199.77**	**1414.98**	**191.89**	**60.67**	**23.25**
SD	1074.61	241.19	44.90	20.00	1.59	1.62
CV%	20.11	2.36	3.17	10.42	2.62	6.95

LLOQ = 1.01 ng/mL

Table 4: Mean brain-to-plasma concentration ratio of Compound-781 following a single intraperitoneal administration to female C57BL/6 mice (Dose: 10 and 30 mg/kg)

Dose (mg/kg)	Time (hr)	Animal ID	Plasma concentration (ng/mL)	Brain concentration (ng/g)	Brain-to-Plasma Ratio	Mean Ratio
10	4	1	114.67	30.93	0.27	0.23
		2	110.18	16.59	0.15	
		3	116.76	32.25	0.28	
	8	4	33.94	11.82	0.35	0.34
		5	29.20	13.08	0.45	
		6	38.41	8.49	0.22	
30	4	7	61.97	0.00	NA	0.09[c]
		8	61.15	5.64	0.09	
		9	58.90	0.00	NA	
	8	10	22.95	36.24	1.58	1.65
		11	21.80	52.23	2.40	
		12	24.99	24.51	0.98	

LLOQ = 3.03 ng/g for brain and 1.01 ng/mL for plasma, NA – Not applicable, c – Single value reported

Figure 1: Mean plasma concentration-time profiles of Compound-781 following a single intraperitoneal dose administration to female C57BL/6 mice (Dose: 10 and 30 mg/kg)

Linear

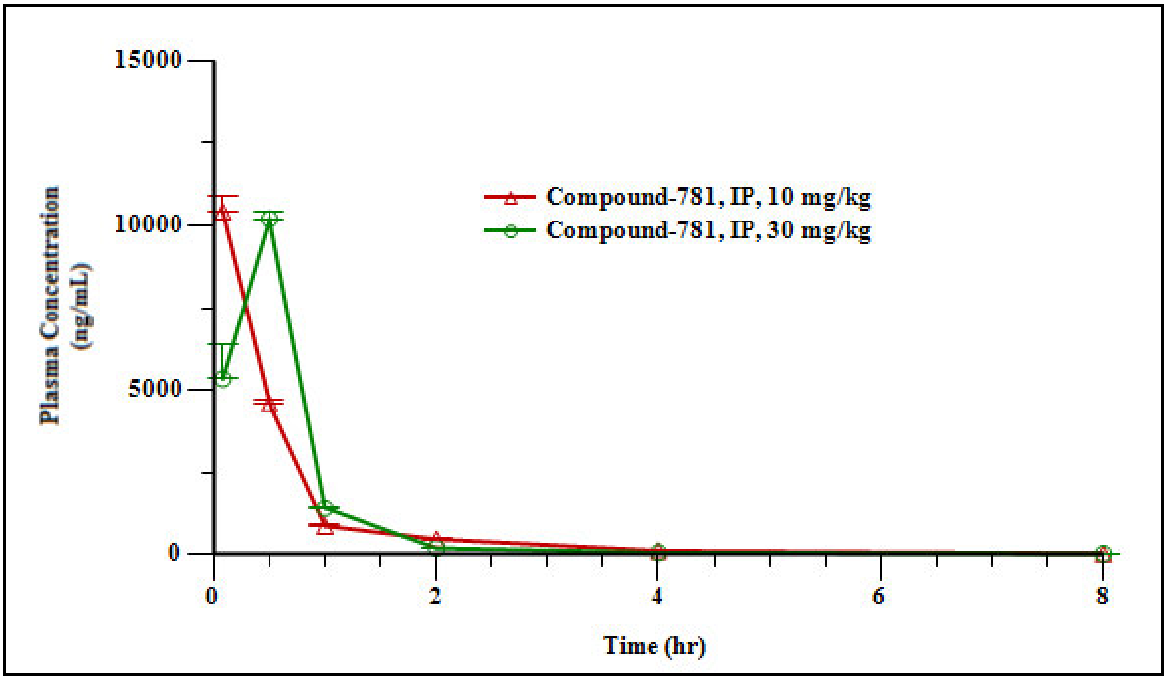

Semi-log

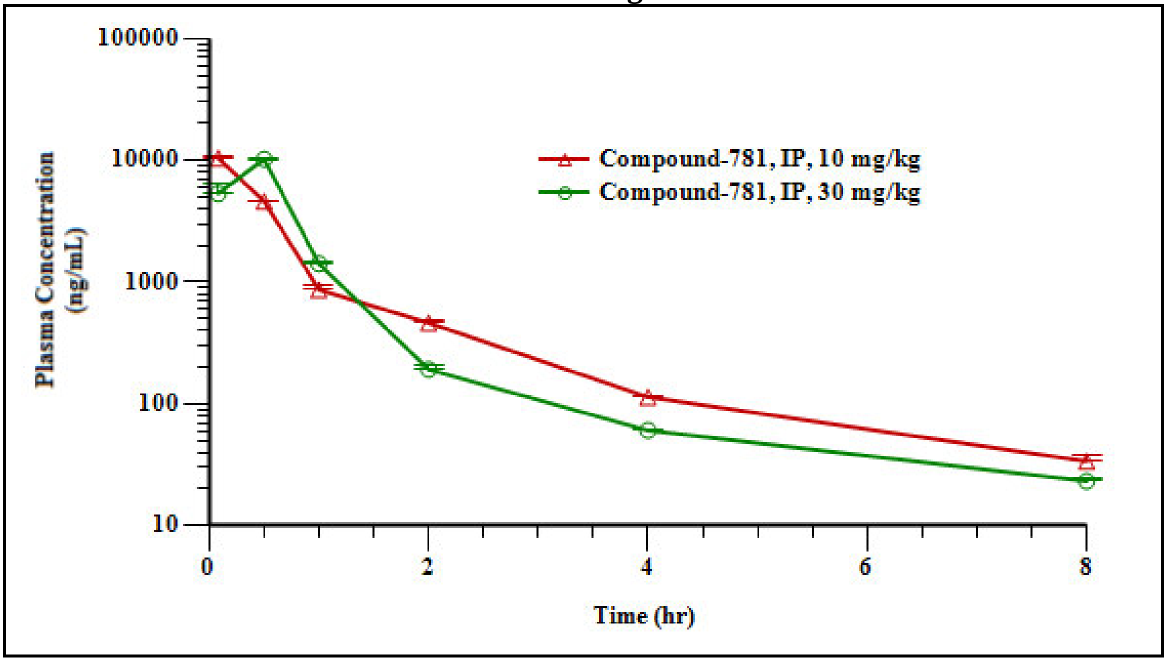

9.0 Annexure I

Bioanalytical Summary

LC Conditions:

Mobile Phase A: 0.1% Formic acid in Acetonitrile

B: 10 Mm Ammonium Formate

Column **:** Accucore Phnyl X, 2.6µm, 50 X 2.1 mm

Injection Volume (µL) **:** 1

Column Oven Temperature (ºC) **:** 45

Retention Time (in min) **: Analyte:** Compound-781 : 0.95

IS: Glipizide: 1.05

LC Gradient Used

Time (Minutes)	Flow Rate (mL/min)	PUMP A (% Conc)	PUMP B (% Conc)
Initial	0.7	0	100
0.30	0.7	0	100
0.50	0.7	95	5
1.30	0.7	95	5
1.40	0.7	0	100
1.80	0.7	0	100

Mass Conditions

MRM Transitions:

Analyte ID / IS ID	Q1	Q3	DP	CE	CXP	Dwell time (msec)
COMPOUND-781 252	516.4	252.2	108	37	18	50
Glipizide	446.3	347.0	40	22	12	50

Source Parameter:

Polarity	Positive
CAD	8
CUR	25
GS1	40
GS2	60
Ion Spray Voltage	5500
Temperature	550
Interface Heater	ON
EP	10

Extraction Procedure:

The extraction procedure for plasma/brain samples and the spiked plasma/brain calibration standards were identical:

A 25 µL of study sample or spiked plasma/brain calibration standard was added to individual pre-labeled micro-centrifuge tubes followed by 100 µL of internal standard prepared in Acetonitrile (Glipizide, 500 ng/mL) was added except for blank, where 100 µL of Acetonitrile was added. Samples were vortexed for 5 minutes. Samples were centrifuged for 10 minutes at a speed of 4000 rpm at 4 °C. Following centrifugation, 100 µL of clear supernatant was transferred in 96 well plates and analyzed using LC-MS/MS.

Representative Chromatograms

Representative chromatogram of blank mice plasma in the LC-MS/MS analysis of COMPOUND-781

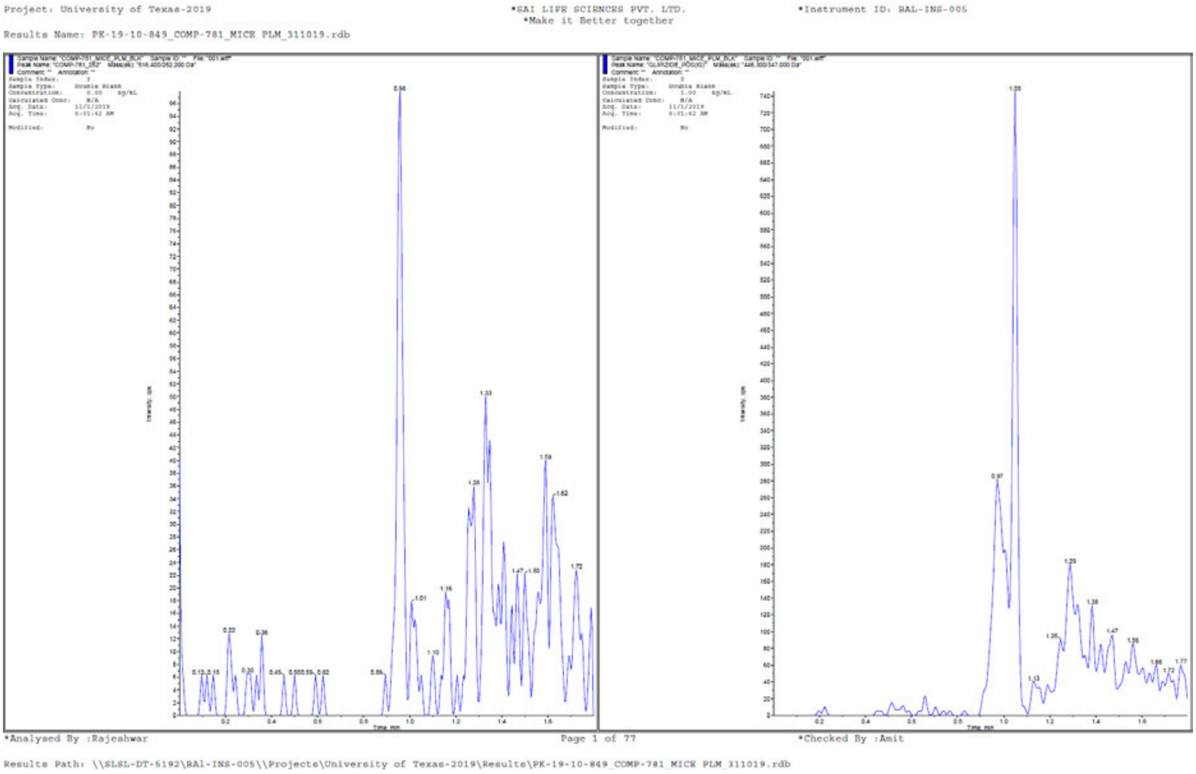

Representative LC-MS/MS chromatogram of COMPOUND-781 LLOQ standard and Glipizide (IS) in mice plasma

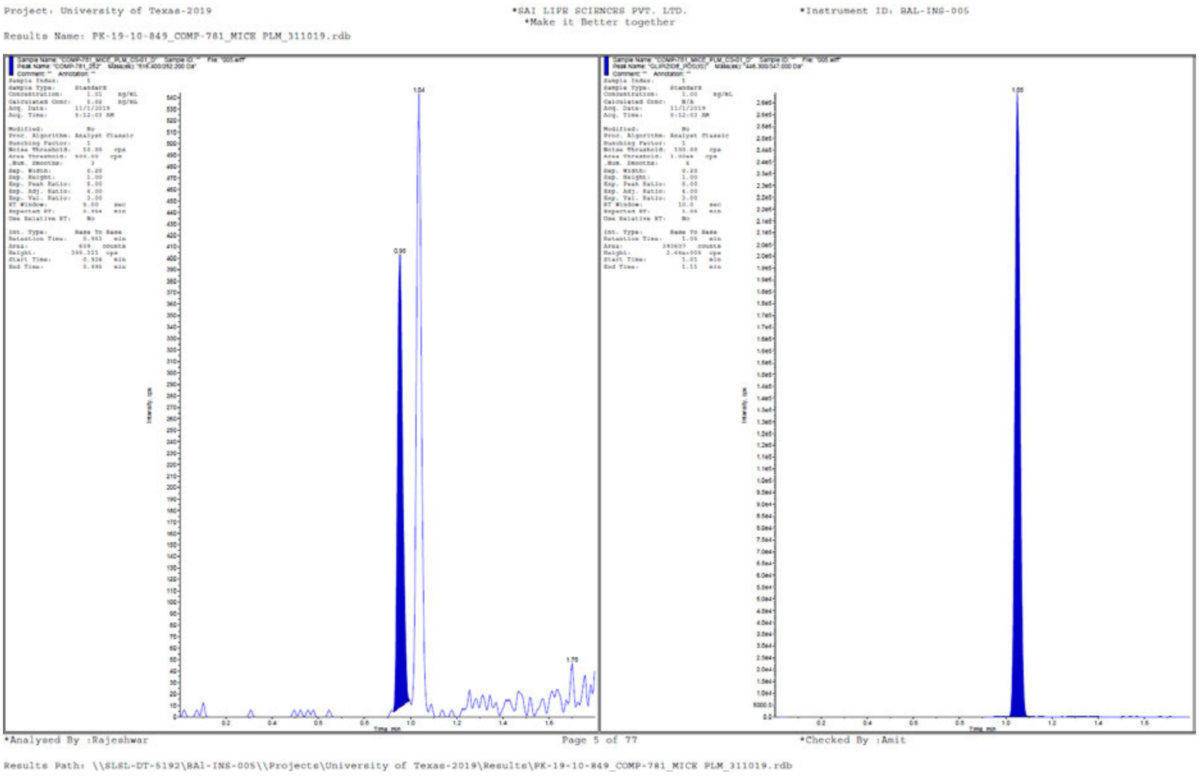

Representative LC-MS/MS chromatogram of COMPOUND-781 ULOQ standard and Glipizide (IS) in mice plasma

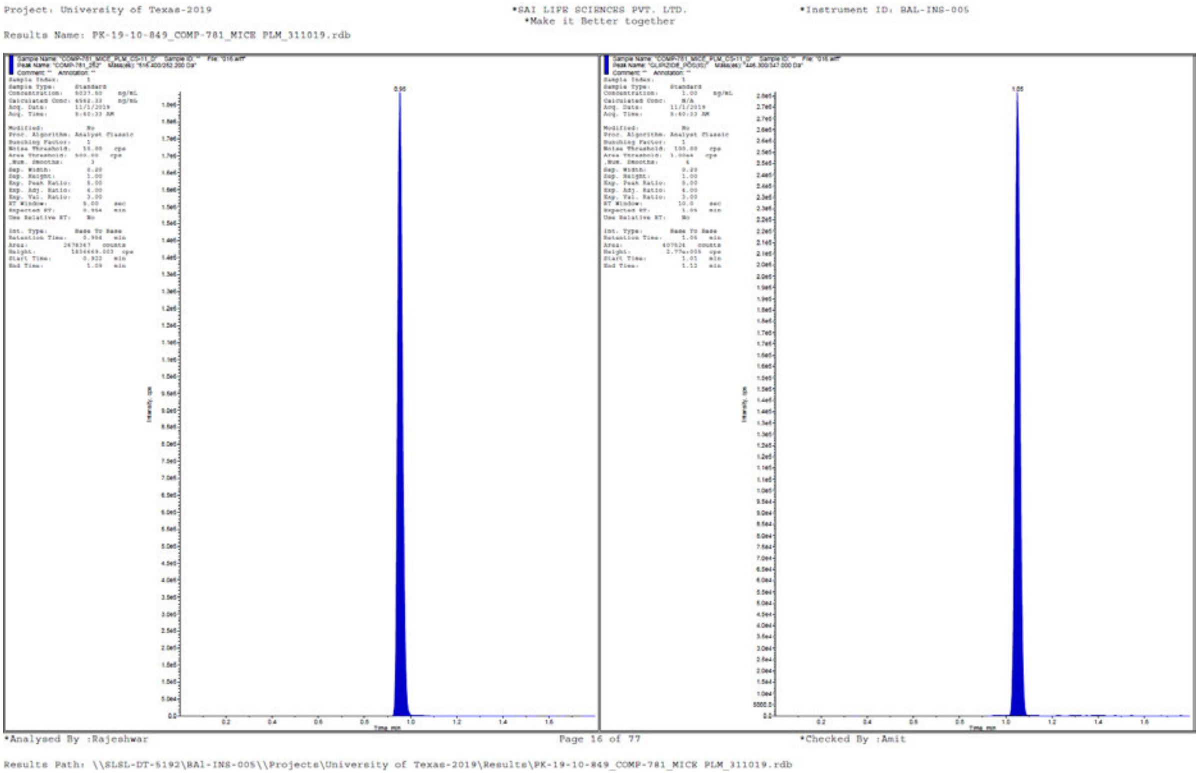

Representative LC-MS/MS chromatogram of COMPOUND-781study sample (plasma) and Glipizide (IS)

Project: University of Texas-2019 *SAI LIFE SCIENCES PVT. LTD. *Make it Better together *Instrument ID: BAL-INS-005

Results Name: PK-19-10-849_COMP-781_MICE PLM_311019.rdb

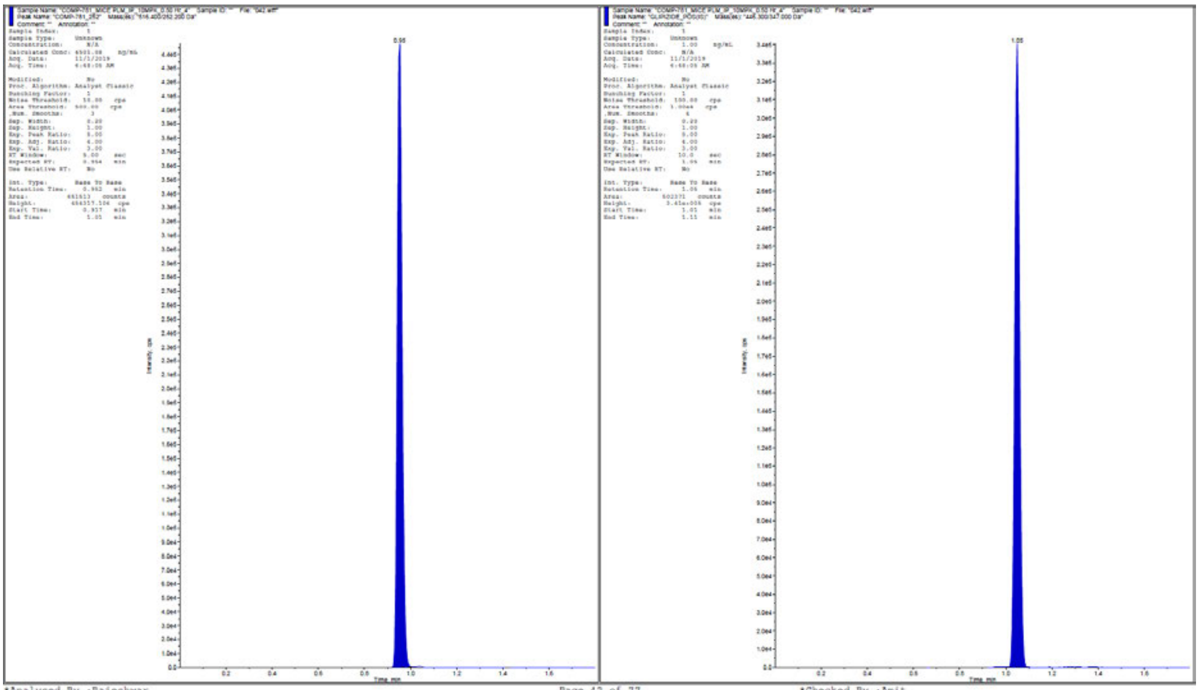

*Analysed By :Rajeshwar Page 42 of 77 *Checked By :Amit

Results Path: \\SLSL-DT-5192\BAI-INS-005\\Projects\University of Texas-2019\Results\PK-19-10-849_COMP-781_MICE PLM_311019.rdb

Calibration curve of COMPOUND-781 in mice plasma

Project: University of Texas-2019 *SAI LIFE SCIENCES PVT. LTD. *Make it Better together *Instrument ID: BAL-INS-005

Results Name: PK-19-10-849_COMP-781_MICE PLM_311019.rdb

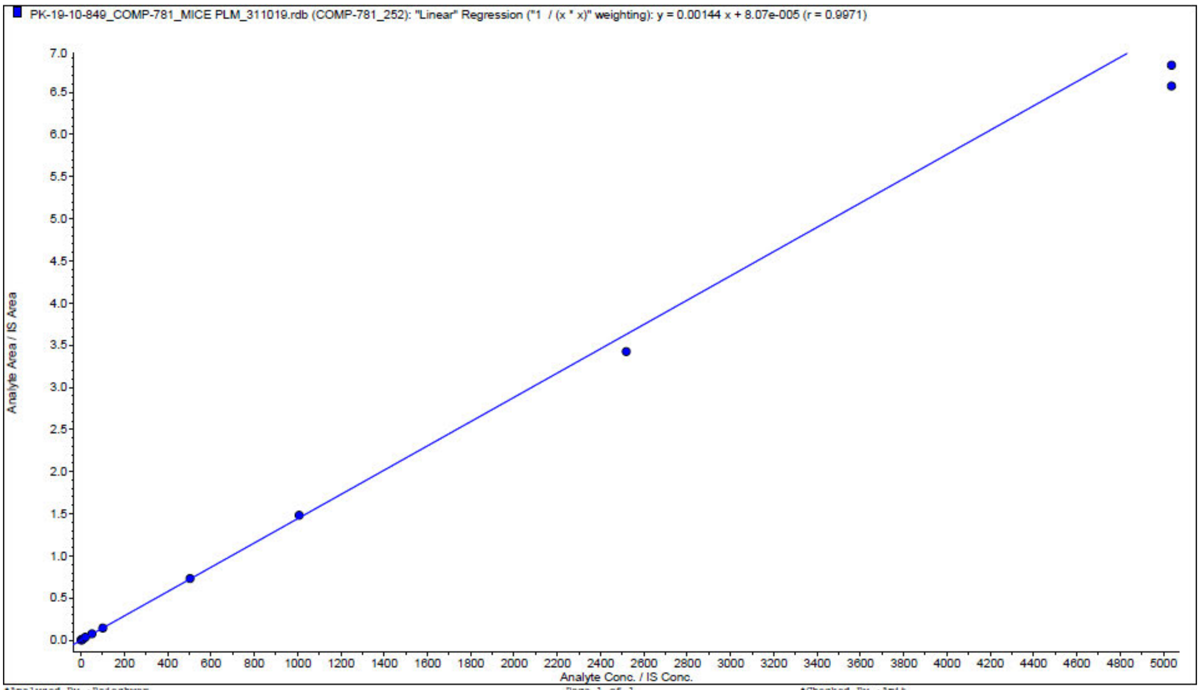

*Analysed By :Rajeshwar Page 1 of 1 *Checked By :Amit

Results Path: \\SLSL-DT-5192\BAI-INS-005\\Projects\University of Texas-2019\Results\PK-19-10-849_COMP-781_MICE PLM_311019.rdb

Calibration curve of COMPOUND-781 in mice brain

Project: University of Texas-2019 *SAI LIFE SCIENCES PVT. LTD. *Make it Better together *Instrument ID: BAL-INS-005

Results Name: PK-19-10-849_COMP-781_MICE BRAIN_311019.rdb

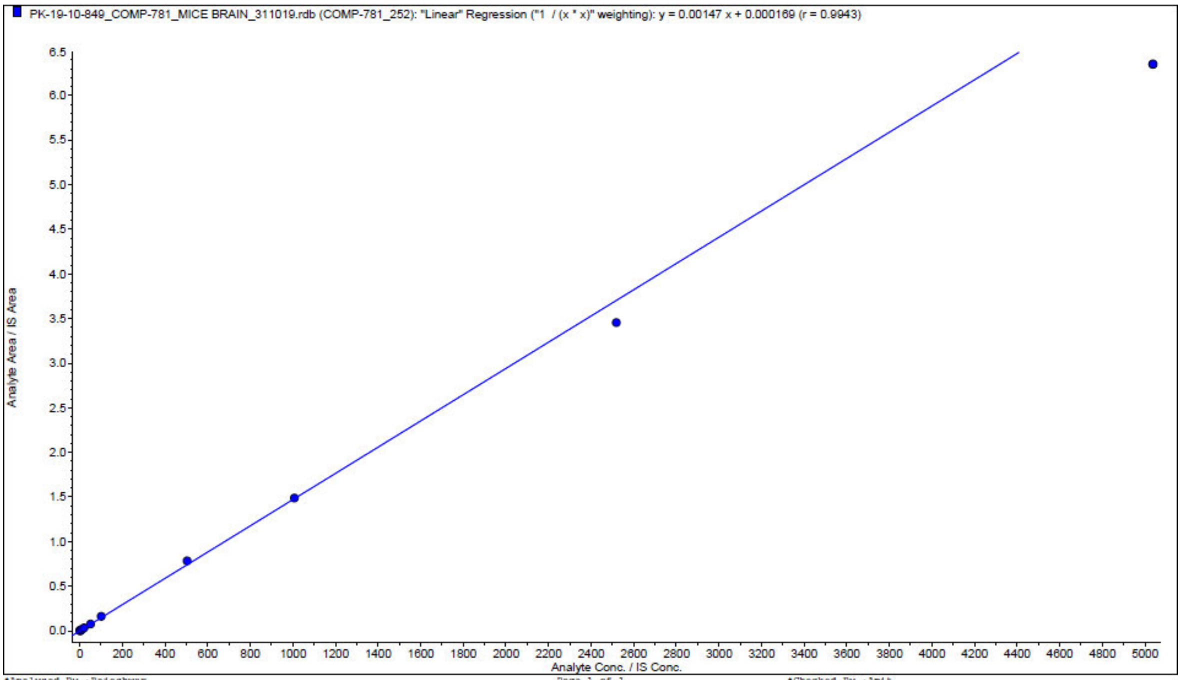

*Analysed By :Rajeshwar Page 1 of 1 *Checked By :Amit

Results Path: \\SLSL-DT-5192\BA1-INS-005\\Projects\University of Texas-2019\Results\PK-19-10-849_COMP-781_MICE BRAIN_311019.rdb

[redacted]
