# Supplementary material for: C781, a β-Arrestin Biased Antagonist at Protease-Activated Receptor-2 (PAR2), Displays in vivo Efficacy Against Protease-Induced Pain in Mice
Source: J Pain. Author manuscript; Available in PMC 2023 Apr 7. (PMC10079573; doi:10.1016/j.jpain.2022.11.006)
Supplement: 3 [file NIHMS1851843-supplement-3.docx]

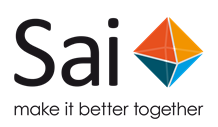


SAI Life Sciences Limited

**METABOLIC STABILITY REPORT**

**Date: 25^th^ November 2019**

**Metabolic stability of Compound-781** **in human and mice liver microsomes**

**Study Number: SAIDMPK/MS-013-11/19**

**Sponsor**

Theodore Price PhD
Eugene McDermott Professor
Director Undergraduate Neuroscience Program
School of Behavioral and Brain Sciences
University of Texas at Dallas
BSB 14.102G
800 W Campbell Rd
Richardson TX 75080
phone: 972-883-4311
cell: 520-471-0360
fax: 972-883-2491

**Testing Facility**

DMPK, Sai Life Sciences Ltd.

Building 1, Plot 2

Chrysalis Enclave

International Biotech Park

Phase II, Hinjewadi

Pune 411 057

INDIA

Phone: +91-20-30125000

**LIST OF ABBREVIATIONS AND SYMBOLS**

| **° C** | : | Degree Centigrade |
| --- | --- | --- |
| **%** | : | Percentage |
| **µL** | : | Microliter |
| **µM** | : | Micromolar |
| **DMSO** | : | Dimethylsulphoxide |
| **pH** | : | Potential of hydrogen |
| **LC-MS/MS** | : | Liquid Chromatography Tandem Mass Spectrometry |
| **mg/mL** | : | Milligram per milliliter |
| **mM** | : | Millimolar |
| **NADPH** | : | β-Nicotinamide Adenine Dinucleotide 2′- Phosphate |
| **+ NADPH** | : | Plus β-Nicotinamide Adenine Dinucleotide 2′- Phosphate |
| **- NADPH** | : | Minus β-Nicotinamide Adenine Dinucleotide 2′- Phosphate |
| **Kphos** | : | Potassium phosphate buffer |
| **G** | : | gram |
| **RPM** | : | Revolutions Per Minute |
| **MRM** | : | Multiple Reaction Monitoring |
| **HLM** | : | Human Liver Microsomes |
| **MLM** | : | Mice Liver Microsomes |
| **min** | : | Minutes |

**CONTENTS**

| **DESCRIPTION** | | | | **Page** |
| --- | --- | --- | --- | --- |
| **TITLE PAGE** | | | | **1** |
| **LIST OF ABBREVIATIONS AND SYMBOLS** | | | | **2** |
| **CONTENTS** | | | | **3** |
| **1.0 Study Responsibilities** | | | | **4** |
| **2.0 Introduction** | | | | **5** |
| **3.0 Study Objective** | | | | **5** |
| **4.0 Study Compliance** | | | | **5** |
| **5.0 Materials** | | | | **5** |
|  | **5.1 Test compounds** | | | **5** |
|  | **5.2 Consumables and reagents** | | | **5** |
|  | **5.3 Equipments** | | | **6** |
| **6.0 Method** | | | | **6** |
|  | **6.1 Preparation of reagents** | | | **6** |
|  |  | | **6.1.1 Preparation of potassium phosphate buffer (50 mM)** | **6** |
|  |  | **6.1.2 Preparation of microsomes** | | **6** |
|  |  | **6.1.3 Preparation of test compounds** | | **6** |
|  |  | **6.1.4 Preparation of NADPH solution** | | **6** |
|  | **6.2 Assay conditions** | | | **6** |
|  | **6.3 Assay** | | | **7** |
|  | **6.4 Bio-analysis** | | | **7** |
| **7.0 Data analysis** | | | | **7** |
| **8.0 Results and Conclusions** | | | | **8** |
| **Tables & Figures** | | | | **9-10** |
| **Annexure 1** | | | | **11-13** |

1. **STUDY RESPONSIBILITIES**

| **Responsibilities** | **Name** |
| --- | --- |
| **Study Director** | **Junaid Farooqui, M.Sc.** |
| **Principal Investigator** | **Sharayu Waghmode, M.Sc.** |
| **Bio-Analysis** | **Gaurav Bhalgat, M.Pharm.** |
| **Report Review** | **Himanshu Rastogi, M.Tech.** |

1. **INTRODUCTION**

Metabolic stability assays using species specific liver microsomes are widely implemented in drug discovery to guide structural modification, predict in vivo performance, develop structure-metabolic stability relationships and triage (sorting) compounds for in vivo animal studies.

1. **STUDY OBJECTIVE**

The objective of the study was to evaluate the metabolic stability of test compound in liver microsomes from human (HLM) and mice (MLM). This was accomplished by incubating the test compound with microsomes and monitoring disappearance with time using LC-MS/MS. Verapamil in HLM and imipramine in MLM were used as positive controls.

1. **STUDY COMPLIANCE**

The study was not performed as per Good Laboratory Practices (GLP) regulations and not audited by Quality Assurance (QA) department. However all appropriate documents are maintained in study file. Data generated and report prepared has been verified for accuracy by study personals.

1. **MATERIALS**
   1. **Test Compound**

Compound-781 was provided by University of Texas in solid form.

- 1. **Consumables and reagents**

| **Materials** | **Catalogue/ Lott No.** | **Manufacturer** |
| --- | --- | --- |
| Pooled human liver microsomes | 452156 | BD Gentest, Woburn, MA |
| Pooled male mice liver microsomes (CD1) | MSMCPL | Gibco |
| Verapamil HCl | V4629 | Sigma, Germany |
| Imipramine hydrochloride | 10899 |  |
| Glipizide | G117 |  |
| Potassium phosphate monobasic | P5655 |  |
| Potassium phosphate dibasic | P2222 |  |
| DMSO | D5879 |  |
| NADPH | 2646-71-1 | SRL India |
| 96 well-plates | PCR-96MR-HS-C | Axygen, Union City, California |

- 1. **Equipments**

| **Equipments** | **Manufacturer** |
| --- | --- |
| Single and multi-channel pipettes | Eppendorf, Germany |
| Thermo-shaker | Grant-bio, England |
| Refrigerated centrifuge | Kubota, Tokyo, Japan |
| LC-MS/MS | Waters ACQUITYTM, ultra-performance LC, Canada |
| API-4000 MDS Sciex | Applied Biosystems, Canada |

1. **METHOD**
   1. **Preparation of reagents**
      1. **Potassium phosphate buffer (kphos)**

50 mM potassium phosphate buffer (pH 7.4) will be prepared by adding 0.647 g potassium phosphate monobasic (KH_2_PO_4_) and 3.527 g potassium phosphate dibasic (K_2_HPO_4_) to 400 mL of Milli-Q water. pH of the buffer will be adjusted to 7.4 and volume will be made up to 500 mL.

- - 1. **Preparation of microsomes**

Microsomes (20 mg/mL) were diluted in Kphos buffer to prepare a concentration of 0.357 mg/mL.

- - 1. **Preparation of test compound**

Stock solution of test compound was prepared in DMSO at a concentration of 1 mM.

- - 1. **Preparation of NADPH solution**

A stock solution of 3.33 mM NADPH (3.33X) was prepared by dissolving appropriate amount of NADPH in Kphos buffer.

- 1. **Assay Conditions**

Total Incubation volume : 100 µL

Compound concentration : 1 µM

Protein Concentration : 0.25 mg/mL

NADPH : 1 mM

Final DMSO contain : 0.1%

Number of replicates : 2

Time points : 0, 5, 15, 30 and 60 min

- 1. **Assay**

An 1120 µL aliquot of Kphos buffer (50 mM, pH 7.4) containing liver microsome (0.357 mg/mL) were added to individual 2 mL tubes (final concentration 0.25 mg/mL). Test and positive control compounds (1 mM DMSO stocks) were directly spiked into respective tubes to prepare a concentration of 1.428 µM (final concentration 1 µM). From the above mix, 70 µL was added to individual wells of 96 well reaction plates and pre-incubated at 37 ^o^C for 5 min. All the reactions were initiated by adding 30 µL of 3.33 mM NADPH (final concentration 1 mM). Reactions without NADPH and buffer controls (minus NADPH) at 0 min and 60 min were also incubated to rule out non-NADPH metabolism or chemical instability in the incubation buffer. All reactions were terminated using 100 µL of ice-cold acetonitrile containing internal standard (glipizide) at 0, 5, 15, 30 and 60 min. The plates were centrifuged at 4000 RPM for 15 min and 100 µL aliquots were submitted for analysis by LC-MS/MS.

- 1. **Bio-Analysis**

Samples were monitored for parent compound disappearance in MRM mode using LC-MS/MS (Annexure 1).

1. **DATA ANALYSIS**
   - The percent remaining of test compound and positive controls in each sample was determined by considering peak area ratio in the 0 minute sample as 100%. The Half-life of compounds in microsomes is calculated by formula:

Half-life (t_1/2_) (min) = 0.693/k, where k is gradient of line determined from plot of peak area ratio (compound peak area / internal standard peak area) against time.

- - Invitro intrinsic clearance (CL’int) (units in mL/min/kg) was calculated using the formula:

$$CL'_{\text{int}} =\frac{0.693}{\text{in vitro T1/2}}.\frac{\text{mL incubation}}{\text{mg microsomes}}. \frac{ 45\text{ mg microsomes} }{\text{gm liver}} .\frac{\text{liver weight in gm*}}{\text{Kg b.w}}$$

For liver microsomes, scaling factor used was 45 mg microsomal protein per gm liver.

* Indicates liver weight (gm) which varies species wise. For human and mice the liver weights are 20 gm and 90 gm respectively.

1. **RESULTS AND CONCLUSIONS**
   - Metabolic stability of positive control compounds verapamil (HLM)) and imipramine (MLM) and used in the experiment were consistent with literature values and validation results generated in-house (Table 1 and figure 1).
   - Metabolic stability results with Compound-781 are provided in Tables 2 and Figures 2.
   - Compound-781 was stable in HLM and MLM within duration of incubation. Details provided in Table 2.

**Table 1**

**Metabolic stability of positive controls in HLM and MLM**

| **Time (min)** | **Verapamil (HLM)** | **Imipramine (MLM)** |
| --- | --- | --- |
| **0** | 100 | 100 |
| **5** | 90 | 88 |
| **15** | 65 | 59 |
| **30** | 37 | 27 |
| **60** | 17 | 9 |
| **% Remaining at 60 min (+ NADPH)** | **17** | **9** |
| **% Remaining at 60 min (- NADPH)** | **111** | **120** |
| **% Remaining at 60 min (Buffer)** | **108** | **109** |
| **t_1/2_ (min)** | **23** | **16** |
| **CL_h,int_ (mL/min/kg)** | **109** | **684** |

**Table 2**

**Metabolic stability of Compound-781 in HLM and MLM**

| **Time (min)** | **HLM** | **MLM** |
| --- | --- | --- |
| **0** | 100 | 100 |
| **5** | 101 | 101 |
| **15** | 99 | 112 |
| **30** | 100 | 99 |
| **60** | 96 | 106 |
| **% Remaining at 60 min (+ NADPH)** | **96** | **106** |
| **% Remaining at 60 min (- NADPH)** | **93** | **101** |
| **% Remaining at 60 min (Buffer)** | **90** | |
| **t_1/2_ (min)** | **>60** | **NC** |
| **CL_h,int_ (mL/min/kg)** | **2** | **NC** |

**NC: Not calculated as compound was stable within the duration of experiment**

**Figure 1**

**Time-dependent loss of positive controls in HLM and MLM**

|  |  |
| --- | --- |

**Figure 2**

**Time-dependent loss of Compound-781** **in HLM and MLM**

|  |  |
| --- | --- |

**Annexure 1**

| **Instrument ID** | API-4000, with Waters UPLC (BAL-INS-001) |
| --- | --- |
|  | |
| **Mobile Phase for** | **A:** 0.1 % Formic acid in Acetonitrile  **B:** 10 mM Ammonium formate |
| **Column** | Kinetex XB, C18, 100A, 50 X 2.1 mm, 1.7 µm |
| **Injection Volume (µL)** | 2 |
| **Column Oven Temperature (ºC)** | 45 |

**LC Gradient Used**

| **Time (min)** | **Flow (mL/min)** | **PUMP A (% Conc.)** | **PUMP B (% Conc.)** |
| --- | --- | --- | --- |
| Initial | 0.800 | 5.0 | 95.0 |
| 0.30 | 0.800 | 5.0 | 95.0 |
| 0.50 | 0.800 | 95.0 | 5.0 |
| 1.40 | 0.800 | 95.0 | 5.0 |
| 1.60 | 0.800 | 5.0 | 95.0 |
| 1.80 | 0.800 | 5.0 | 95.0 |

**Retention time of Analyte**

| **Analyte** | **Retention time (min)** |
| --- | --- |
| COMP-781 | 0.71  71 |
| IMIPRAMINE | 0.78 |
| VERAPAMIL | 0.78 |

**Mass Conditions**

**MRM Transitions**

| **Analyte ID / IS ID** | **Q1** | **Q3** | **DP** | **CE** | **CXP** | **Dwell time (msec)** |
| --- | --- | --- | --- | --- | --- | --- |
| COMP-781 | 516.400 | 252.200 | 108 | 37 | 18 | 50 |
| VERAPAMIL | 455.5 | 165.5 | 105 | 42 | 11 | 50 |
| IMIPRAMINE | 281.3 | 86 | 102 | 25 | 16 | 50 |
| Glipizide | 446.3 | 347.0 | 40 | 22 | 12 | 50 |

**Source Parameter Used Positive Polarity**

| **Source Parameter** | **Parameter value/ description** |
| --- | --- |
| Polarity | Positive |
| CAD | 8 |
| CUR | 25 |
| GS1 | 40 |
| GS2 | 60 |
| Ion Spray Voltage | 5500 |
| Temperature | 550 |
| Interface Heater | ON |
| EP | 10 |

**Representative Chromatograms: Provided below**

**Figure 1: Representative LC-MS/MS chromatogram of COMP-781**

**Figure 2: Representative LC-MS/MS chromatogram of IMIPRAMINE**

**Figure 3: Representative LC-MS/MS chromatogram of VERAPAMIL**
