# Supplementary material for: C781, a β-Arrestin Biased Antagonist at Protease-Activated Receptor-2 (PAR2), Displays in vivo Efficacy Against Protease-Induced Pain in Mice
Source: J Pain. Author manuscript; Available in PMC 2023 Apr 7. (PMC10079573; doi:10.1016/j.jpain.2022.11.006)
Supplement: 4 [file NIHMS1851843-supplement-4.docx]

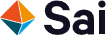


SAI Life Sciences Limited

**PLASMA PROTEIN BINDING REPORT**

**Date: 26^th^ November 2019**

**Determination of protein binding of Compound-781** **in human and mice plasma using rapid equilibrium dialysis method**

**Study Number: SAIDMPK/PPB-008-11/19**

**Sponsor**

Theodore Price PhD
Eugene McDermott Professor
Director Undergraduate Neuroscience Program
School of Behavioral and Brain Sciences
University of Texas at Dallas
BSB 14.102G
800 W Campbell Rd
Richardson TX 75080
phone: 972-883-4311
cell: 520-471-0360
fax: 972-883-2491

**Testing Facility**

DMPK, Sai Life Sciences Ltd.

Building 1, Plot 2

Chrysalis Enclave

International Biotech Park

Phase II, Hinjewadi

Pune 411 057

INDIA

Phone: +91-20-30125000

**LIST OF ABBREVIATIONS AND SYMBOLS**

| **° C** | : | Degree Centigrade |
| --- | --- | --- |
| **%** | : | Percentage |
| **µL** | : | Microliter |
| **µM** | : | Micromolar |
| **pH** | : | Potential of hydrogen |
| **DMSO** | : | Dimethylsulphoxide |
| **LC-MS/MS** | : | Liquid Chromatography Tandem Mass Spectrometry |
| **mM** | : | Millimolar |
| **RPM** | : | Revolutions Per Minute |
| **MRM** | : | Multiple Reaction Monitoring |

**CONTENTS**

| **DESCRIPTION** | | **PAGE** |
| --- | --- | --- |
| **TITLE PAGE** | | **1** |
| **LIST OF ABBREVIATIONS AND SYMBOLS** | | **2** |
| **CONTENTS** | | **3** |
| **1.0 Study Responsibilities** | | **4** |
| **2.0 Introduction** | | **5** |
| **3.0 Study Objective** | | **5** |
| **4.0 Materials** | | **5** |
|  | **4.1 Test compound** | **5** |
|  | **4.2 Consumables and reagents** | **5** |
|  | **4.3 Equipments** | **6** |
| **5.0 Method** | | **6** |
|  | **5.1 Assay Condition** | **6** |
|  | **5.2 Preparation of Reagents** | **6** |
|  | **5.2 Assay procedure** | **6** |
|  | **5.3 Sample preparation and bio-analysis** | **7** |
| **6.0 Data analysis** | | **7** |
| **7.0 Results and Conclusions** | | **7** |
| **Table** | | **8** |

1. **STUDY RESPONSIBILITIES**

| **Responsibilities** | **Name** |
| --- | --- |
| **Study Director** | **Junaid Farooqui, M.Sc.** |
| **Principal Investigator** | **Sharayu Waghmode, M.Sc.** |
| **Bio-Analysis** | **Rajeshwar Jalnapurkar, M.Pharm.** |
| **Report Review** | **Himanshu Rastogi, M.Tech** |

1. **INTRODUCTION**

In drug discovery, the information on drug–plasma protein binding is valuable to evaluate and better understand absorption, distribution, metabolism and excretion (ADME) related properties and the pharmacokinetic profile of drug candidates. It is widely believed that only the free concentration rather than the total drug concentration is pharmacologically active. Plasma protein binding (PPB) data are useful to design optimal dose regimens for efficacy studies and to estimate safety margins during drug development. Many lead molecules with high affinities for a therapeutic target in vitro exhibit a reduced efficacy in vivo. Therefore, the determination of the free fraction (unbound drug) of the drug becomes one of the important issues for both in vitro and in vivo screening of potential drug candidates. Several methods have been applied for the measurement of plasma protein binding including equilibrium dialysis, ultrafiltration and ultracentrifugation combined with LC-MS/MS. Currently, equilibrium dialysis is the most widely used methods for protein binding measurements. Recently, rapid equilibrium dialysis is being applied to determine the protein binding in early drug discovery as several compounds can be evaluated in a high throughput screening mode.

1. **STUDY OBJECTIVE**

The objective of this study was to evaluate protein binding test compounds in human and mice plasma. This was accomplished by spiking the test compounds at a concentration of 5 µM into human and mice plasma and dialyzing against buffer until equilibrium is achieved (4 hours). Analyte area ratios of the test compounds in plasma and buffer were determined to calculate unbound and bound percentages of compound to the plasma proteins.

1. **MATERIALS**
   1. **Test Compounds**

Compound-781 was provided by University of Texas in solid form.

- 1. **Consumables and reagents**

| **Materials** | **Catalog/ Lott No.** | **Manufacturer** |
| --- | --- | --- |
| Warfarin | 376-34A | Supelco, West Chester, PA |
| DMSO | D5879 | Sigma, Germany. |
| Human plasma (drug free volunteers; n = 6) | Not applicable | Poona Research Foundation, India |
| Mice plasma (CD-1 ) | Not applicable | Collected fresh in-house |
| RED device inserts | 89809 | Thermo Scientific, Rockford, IL |
| Phosphate buffered saline pH 7.4 | JF1134861 | Thermo Scientific, Rockford, IL |

- 1. **Equipments**

| **Equipments** | **Manufacturer** |
| --- | --- |
| Single and multi-channel pipettes | Eppendorf, Germany |
| Refrigerated centrifuge | Kubota, Tokyo, Japan |
| RED device base plate | Thermo Scientific, Meridian Rd., Rockford, IL |
| Orbital shaker | Heidolph Instruments, Schwabach, Germany |
| CO2 incubator | Thermo Scientific, 81 Wyman Street, Waltham, MA |
| LC-MS/MS | Waters ACQUITYTM, ultra performance LC, Canada |
| API-4000 MDS Sciex | Applied Biosystems, Canada |

1. **METHOD**
   1. **Assay conditions**

Compound concentration : 5 µM

Matrix : human and mice plasma

Incubation temperature : 37^o^C

Incubation time : 4 hrs

DMSO : 0.5%

Replicates : 3

- 1. **Preparation of Reagents**

A 1 mM stock solutions test compound were prepared in DMSO and diluted 200-folds in human and mice plasma to prepare a concentration of 5 µM. The final DMSO concentration in plasma was 0.5%.

- 1. **Assay Procedure**

Rapid equilibrium dialysis was performed with a rapid equilibrium dialysis (RED) device containing dialysis membrane with a molecular weight cut-off of 8,000 Daltons. Each dialysis insert contains two chambers. The red chamber is for plasma while the white chamber is for buffer.

A 200 µL aliquot of warfarin and test compound at 5 µM (triplicates) were separately added to the plasma chamber and 350 µL of phosphate buffer saline (pH 7.4) was added to the buffer chamber of the inserts. After sealing the RED device with an adhesive film, dialysis was performed in incubator at 37 ^0^C with shaking at 100 RPM for 4 hours.

*Recovery and stability:* A 50 µL aliquot of warfarin and test compounds were added to four 0.5 mL microfuse tubes. Two aliquots were frozen immediately (0 minute sample). The other two aliquots were incubated at 37 ^o^C for 4 hours along with the RED device.

Following dialysis, an aliquot of 50 µL was removed from each well (both plasma and buffer side) and diluted with equal volume of opposite matrix (dialyzed with the other matrix) to nullify the matrix effect. Similarly, 50 µL of buffer was added to recovery and stability samples. An aliquot of 100 µL was submitted for LC-MS/MS analysis.

- 1. **Sample preparation and Bio-analysis**

A 25 µL aliquot of warfarin and test compounds were crashed with 100 µL of acetonitrile containing internal standard (glipizide) and vortexed for 5 minutes. The samples were centrifuged at 4000 RPM at 4 ^0^C for 10 min and 100 µL of supernatant was submitted for LC-MS/MS analysis. Samples were monitored for parent compound in MRM mode using LC-MS/MS. The LC-MS/MS conditions and MRM chromatogram will be provided as per clients request.

1. **DATA ANALYSIS**

The peak area ratios (analyte versus internal standard) obtained was used to determine the fraction of compound bound to plasma proteins. The following equation was used to determine the extent of plasma protein binding:

Percent free drug = 100 x (peak area ratio in dialysate buffer)

(Peak area ratio in dialysate plasma)

Percent bound drug = 100 - % free drug

***Recovery (%):*** 100 x (response _plasma_ + response _buffer_) after dialysis / (response _plasma_) incubated at 37 ºC in tubes along with RED device.

***Stability (%):***100 x (response _plasma_) incubated at 37 ºC in tubes along with RED device / (response _plasma_) samples frozen at 0 min

1. **RESULTS AND CONCLUSIONS**

- Plasma protein binding (PPB) of positive control warfarin used in the study is consistent with data reported in literature and validation results generated in-house (Table 1)
- The percentage binding in human and mice plasma for test compounds is represented in Table 1.
- Compound-781 show moderate binding 67.7 % and 60.7 % in human and mice plasma represented in Table 1.

**Table 1**

**Plasma protein binding of warfarin and test compounds in human and mice plasma**

| **Compounds** | **Species** | **% Bound** | | | | **% Recovery** | **% compound remaining at**  **4 hrs** |
| --- | --- | --- | --- | --- | --- | --- | --- |
|  |  | **R1** | **R2** | **R3** | **Mean ± SD** |  |  |
| **Warfarin** | Human | 98.8 | 99.0 | 98.9 | 98.9 ± 0.1 | 97 | 100 |
|  | Mice | 94.2 | 94.3 | 93.0 | 93.9 ± 0.7 | 94 | 101 |
| **Compound-781** | Human | 69.4 | 64.7 | 69.0 | 67.7 ± 2.6 | 87 | 114 |
|  | Mice | 64.7 | 63.7 | 53.7 | 60.7 ± 6.0 | 89 | 94 |
