# Supplementary material for: C781, a β-Arrestin Biased Antagonist at Protease-Activated Receptor-2 (PAR2), Displays in vivo Efficacy Against Protease-Induced Pain in Mice
Source: J Pain. Author manuscript; Available in PMC 2023 Apr 7. (PMC10079573; doi:10.1016/j.jpain.2022.11.006)

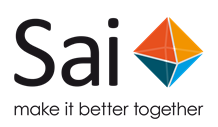


Sai Life Sciences Limited

**AQUEOUS SOLUBILITY REPORT**

**Date: 26^th^ November 2019**

**Aqueous solubility of compound-781** **at pH 2.0 and 7.4**

**Study Number: SAIDMPK/ASA-013-11/19**

**Sponsor**

Theodore Price PhD
Eugene McDermott Professor
Director Undergraduate Neuroscience Program
School of Behavioral and Brain Sciences
University of Texas at Dallas
BSB 14.102G
800 W Campbell Rd
Richardson TX 75080
phone: 972-883-4311
cell: 520-471-0360
fax: 972-883-2491

**Testing Facility**

DMPK, Sai Life Sciences Ltd.

Building 1, Plot 2,

Chrysalis Enclave,

International Biotech Park,

Phase II, Hinjewadi,

Pune 411 057

INDIA

Phone: +91-20-66743600

Fax: +91-20-667436

**LIST OF ABBREVIATIONS AND SYMBOLS**

| **%** | : | Percentage |
| --- | --- | --- |
| **µL** | : | Microliter |
| **µM** | : | Micromolar |
| **nm** | : | Nanometer |
| **mM** | : | Millimolar |
| **N** | : | Normal |
| **λmax** | : | Lambda max |
| **DMSO** | : | Dimethylsulphoxide |
| **RPM** | : | Revolutions Per Minute |
| **pH** | : | Potential of hydrogen |
| **OD** | : | Optical Density |
| **UV** | : | Ultraviolet |
| **SS** | : | System Solution |
| **NaOH** | : | Sodium hydroxide |
| **HCl** | : | Hydrochloric acid |

**CONTENTS**

| **DESCRIPTION** | | **Page** |
| --- | --- | --- |
| **TITLE PAGE** | | **1** |
| **LIST OF ABBREVIATIONS AND SYMBOL** | | **2** |
| **CONTENTS** | | **3** |
| **1.0 Study Responsibilities** | | **4** |
| **2.0 Introduction** | | **5** |
| **3.0 Study Objective** | | **5** |
| **4.0 Materials** | | **5** |
|  | **4.1 Test compound** | **5** |
|  | **4.2 Consumables and reagents** | **5** |
|  | **4.3 Equipment** | **5** |
| **5.0 Method** | | **6** |
|  | **5.1 Preparation of reagents** | **6** |
|  | **5.2 Assay conditions** | **6** |
|  | **5.3 Solubility Assay Procedure** | **6** |
| **6.0 Data Analysis** | | **7** |
| **7.0 Results and Conclusions** | | **7** |
| **Tables & Figures** | | **8-10** |

1. **STUDY RESPONSIBILITIES**

| **Study Director** | **Tufan Tarade, M.Pharm.** |
| --- | --- |
| **Principal Investigator** | **Pradnya Honrao, M.Sc.** |
| **Technical Coordinators** | **Pradnya Honrao, M.Sc.** |
| **Report Review** | **Himanshu Rastogi, M.Tech.** |

1. **INTRODUCTION**

The aqueous solubility of a New Chemical Entity (NCE) is one of the key Physico-Chemical properties that affect its absorption through the gastrointestinal tract following oral administration. Therefore, determination of aqueous solubility at different pH is invaluable in the selection of the most promising potential drug candidates.

1. **STUDY OBJECTIVE**

The objective of the study was to assess the aqueous solubility of test compound in system solution (SS) at pH 2.0 and 7.4. This was accomplished by spiking known concentration of test compound (dissolved in DMSO) into SS, followed by incubation for 4 hours. Optical density of the test compound and reference were measured at the end of incubation to determine solubility. Glipizide was run as a positive control in this study.

1. **MATERIALS**
   1. **Test Compound**

Compound-781 was provided by University of Texas.

- 1. Consumables and reagents

| **Materials** | **Catalog/ Lott No.** | **Manufacturer** |
| --- | --- | --- |
| System solution (SS) | P/N 110151 | *p*ION, Woburn, MA |
| DMSO-GC grade, 99.5% purity | 038K0710 | Sigma |
| Glipizide | G117 |
| Isopropanol - GC grade, 99.9% purity | 0109127 | Spectrochem, Mumbai, India |
| 96 deep well plates | P/N: 110023 | pION, Woburn, MA |
| Filtration plates | P/N:110037 |
| UV plates | P/N: 110286 |

- 1. **Equipment**

| **Equipments** | **Manufacturer** |
| --- | --- |
| Single and multi-channel pipettes | Eppendorf, Germany |
| Spectra Max Plus - Version 2.1000 | Molecular Devices, Sunnyvale, CA |
| Orbital Shaker | Heidolph, Germany |

1. **METHOD**
   1. **Preparation of reagents**
      1. **1X System Solution (SS)**

SS (phosphate free buffer) was supplied as 40X concentrate (pH 2.6). The concentrate was diluted 40-folds with Milli-Q water and pH was adjusted to 2.0 using 2.0 N HCl and pH was adjusted to 7.4 using 0.5 N NaOH.

- - 1. **Test compounds and positive control**

A 20 mM stock solution of test compound and positive control was prepared in DMSO.

- 1. **Assay Conditions**

Total Incubation volume : 500 µL

Compound concentration : 200 µM

pH conditions : 2 and 7.4

Incubation time : 4 hours

Number of replicates : 3

Final DMSO contain : 1%

- 1. **Solubility Assay Procedure**

In this assay, essentially three plates were used, blank plate, reference plate and a sample plate. The blank plate contains no test or reference compounds. The reference plate contains both test compounds and positive control compounds, but dissolved in propanol to obtain 100% reference values for solubility. The third plate (sample plate) contains both test compounds and positive control compounds for aqueous solubility measurement. A flow diagram (Figure 2) shows a sequential procedure for solubility analysis. Blank, reference and sample solutions were prepared and measured in sequence as mentioned below.

- - 1. **Blank Plate**

Blank plate was prepared by adding 5 µL of DMSO to 95 µL of propanol. Further 75 µL aliquot of SS were mixed with 70 µL of propanol and 5 µL of the above stock and scanned between 190-400 nm using a spectrophotometer

- - 1. **Reference Plate**

Reference stock plate of test and positive control compounds was prepared by adding 5 µL of 20 mM to 95 µL of propanol (propanol ensures that the compound stays in solution). Further 75 µL aliquot of SS were mixed with 70 µL of propanol and 5 µL of the above stock and scanned between 190-400 nm.

- - 1. **Sample Plate**

A 20 mM stock of test compound and positive controls were diluted 100-folds in 1X system solution to attain a final working concentration of 200 µM. The percentage of DMSO in the final incubation was ensured to be ≤ 1%, to minimize DMSO effect on solubility of test and positive control samples. A 500 µL aliquot of the diluted test compounds and positive controls, were added to 96 deep well plates in triplicates. The plates were sealed and incubated at room temperature for 4 hours with constant shaking (250 RPM). After incubation, 300 µL of sample was taken from the deep well incubation plate and filtered using *p*ION filter plates. A 75 µL aliquot of the filtered sample was mixed with 75 µL of propanol and scanned using the spectrophotometer between 190-400 nm.

1. **DATA ANALYSIS**

Optical density at λ_max_ was taken to measure solubility. Solubility in micro molar concentration was calculated using the following formula:

Aqueous Solubility (µM) = 150/75*(OD of Sample/OD of reference)*C_R_.

C_R_: Concentration of reference (33.33 µM).

1. **RESULTS AND CONCLUSIONS**

- Solubility results of positive control compound Glipizide at pH 2.0 and 7.4 were consistent with previously generated in-house validation results (Table 1 and Figure 1).
- Solubility of Compound-781 is represented in Table 1 and Figure 1.

**Table 1**

**Aqueous solubility of Glipizide (positive control) and Compound-781 at pH 2.0 and 7.4**

| **Compounds** | **Wavelength (nm)** | **Mol. Wt.** | **Solubility at**  **pH 2.0 (µM)** | **Solubility at**  **pH 7.4 (µM)** |
| --- | --- | --- | --- | --- |
| **Glipizide** | 260 | 445.5 | <5 | 130.8 ± 1.2 |
| **Compound-781** |  | 515.0 | 174.7 ± 2.8 | 162.6 ± 8.6 |

**Figure 1**

**Aqueous solubility of Glipizide (positive control) and Compound-781 at pH 2.0 and 7.4**

**Figure 2**

**Flow diagram representing solubility analysis**


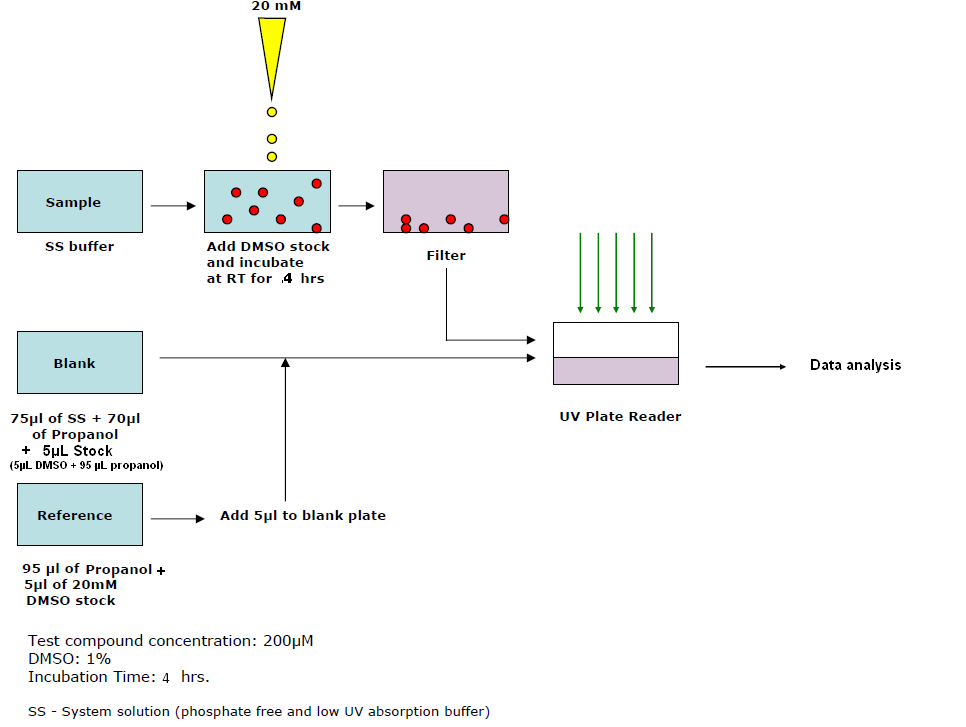

Supplement: 5 [file NIHMS1851843-supplement-5.doc]
